# Synthesis of a Redox-Active NNP-Type Pincer Ligand and Its Application to Electrocatalytic CO_2_ Reduction With First-Row Transition Metal Complexes

**DOI:** 10.3389/fchem.2019.00330

**Published:** 2019-05-21

**Authors:** Kallol Talukdar, Asala Issa, Jonah W. Jurss

**Affiliations:** Department of Chemistry and Biochemistry, University of Mississippi, University, MS, United States

**Keywords:** molecular catalyst, CO_2_ reduction, electrocatalysis, pincer ligand, first-row metals

## Abstract

We report the synthesis of a rigid phosphine-substituted, redox-active pincer ligand and its application to electrocatalytic CO_2_ reduction with first-row transition metal complexes. The tridentate ligand was prepared by Stille coupling of 2,8-dibromoquinoline and 2-(tributylstannyl)pyridine, followed by a palladium-catalyzed cross-coupling with HPPh_2_. Complexes were synthesized from a variety of metal precursors and characterized by NMR, high-resolution mass spectrometry, elemental analysis, and cyclic voltammetry. Formation of bis-chelated metal complexes, rather than mono-chelated complexes, was favored in all synthetic conditions explored. The complexes were assessed for their ability to mediate electrocatalytic CO_2_ reduction, where the cobalt complex was found to have the best activity for CO_2_-to-CO conversion in the presence of water as an added proton source.

## Introduction

Carbon dioxide (CO_2_) is a greenhouse gas which is produced in large quantities from fossil fuel combustion. From the Industrial Revolution forward, anthropogenic CO_2_ emissions have increased at an alarming rate, along with associated concerns including climate change, rising sea levels, and ocean acidification (Hansen et al., 2008; Jiang and Guan, 2016; Blunden and Arndt, 2017). These environmental issues can be mitigated by effective technologies capable of converting CO_2_ into sustainable fuels (Lim et al., 2014). In this context, electrocatalytic CO_2_ reduction to generate carbon-neutral fuels and value-added commodity chemicals is a promising strategy that allows CO_2_ to serve as a cheap and abundant C_1_ feedstock.

**Figure 1:**
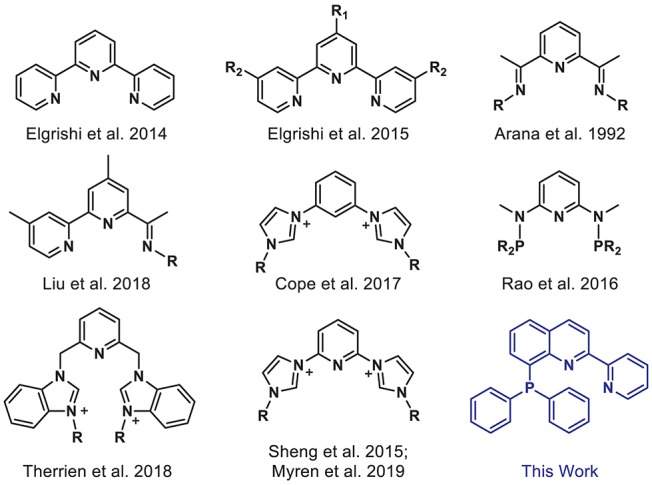
Selected pincer ligands used with first-row transition metals for electrocatalytic CO_2_ reduction.

However, reducing CO_2_ is an energetically uphill process, and more efficient and selective catalysts are needed to facilitate its conversion. Many homogeneous metal complexes have been developed for CO_2_ reduction, but these systems often rely on precious metals and/or are limited to privileged supporting ligands such as tetraazamacrocycles and bipyridines (Qiao et al., 2013; Francke et al., 2018). Tridentate “pincer” ligands represent an underexplored class of ligands, which are of particular interest given their strong chelate effect, preorganized geometry, high tunability, and potential for ligand-based redox activity (van der Vlugt and Reek, 2009; Younus et al., 2014; Peris and Crabtree, 2018). Indeed, Earth-abundant first-row transition metals supported by well-designed pincer ligands have been successfully employed in a variety of catalytic applications (Benito-Garagorri and Kirchner, 2008; van der Vlugt and Reek, 2009; van der Vlugt, 2012; Chakraborty et al., 2015; Bauer and Hu, 2016). Yet, only a limited number of pincer complexes have been reported for electrocatalytic CO_2_ reduction (Arana et al., 1992; Chiericato et al., 2000; Elgrishi et al., 2014, 2015; Sheng et al., 2015; Rao et al., 2016; Cope et al., 2017; Liu et al., 2018; Therrien et al., 2018; Myren et al., 2019). Early examples from Arana et al. (1992) and Chiericato et al. (2000) were reported with redox-active terpyridine and bis(imino)pyridine-based ligands (Figure 1). The metal ions decorated with these NNN-type ligands showed catalytic activity under reducing potentials in CO_2_-saturated solutions. Detailed product analysis was not reported, but formic acid was a notable reduced carbon product obtained with a cobalt bis(imino)pyridine complex. Recently, Liu et al. (2018) prepared a related pincer ligand, based in part on bis(imino)pyridine, in which steric bulk was added and one arm was replaced with pyridine to access a bipyridyl unit, producing a cobalt catalyst that is selective for CO_2_ reduction to formate. Rao et al. (2016) reported a PNP-ligated manganese-tricarbonyl complex which can reduce CO_2_ to CO without any added Brønsted acid. Mixed-donor *N*-heterocyclic carbene-based pincer ligands have also proven to be effective supporting frameworks for transition metal catalysts for CO_2_ conversion (Sheng et al., 2015; Cope et al., 2017; Therrien et al., 2018; Myren et al., 2019).

Given these results, we sought to design a new redox-active mixed-donor ligand with extended conjugation for CO_2_ reduction employing first-row transition metals. The ligand design aims to exploit the well-established redox activity of bipyridine at a lower overpotential by extending its conjugation. In addition, the triphenylphosphine donor fragment serves as a good π-acceptor to facilitate metal-based reductions at lower potentials. We note that closely-related NNP ligands have been recently reported (Basu et al., 2018; Kamitani et al., 2018) and applied to the synthesis of new Fe and Co complexes for catalytic hydrosilylation and dehydrogenative silylation. Herein, first-row metal complexes of a rigid NNP pincer ligand and their electrocatalytic activity toward CO_2_ reduction are reported.

## Experimental

### Materials and Methods

All synthetic manipulations were carried out using standard Schlenk techniques or in an MBraun glovebox under nitrogen atmosphere. Freshly distilled acetonitrile (CH_3_CN) and *N,N*-dimethylformamide (DMF) were used in synthesis and electrochemistry. Tetrahydrofuran, toluene, and diethyl ether were dried with a Pure Process Technology solvent purification system. 1,1′-Bis(diisopropylphosphino)-ferrocene (DiPPF), diphenylphosphine, iron(II) trifluoromethanesulfonate, and copper(II) perchlorate hexahydrate were purchased from Strem Chemicals. Zinc(II) trifluoromethanesulfonate and sodium tert-butoxide were purchased from Acros Organics. Nickel perchlorate hexahydrate and palladium(II) acetate were purchased from Alfa Aesar and Chem-Impex International, respectively. All other chemicals were reagent or ACS grade, purchased from commercial vendors, and used without further purification. ^1^H, ^31^P, and ^13^C NMR spectra were obtained using Bruker spectrometers operating at 500 MHz (^1^H), 167 MHz (^31^P), or 126 MHz (^13^C). Spectra were calibrated vs. observed solvent peaks. Chemical shifts are reported in parts per million (ppm). Solution magnetic susceptibilities were determined by NMR using the Evans method. High-resolution electrospray ionization mass spectra (HR-ESI-MS) were obtained with a Waters SYNAPT HDMS Q-TOF mass spectrometer and elemental analyses of carbon, hydrogen, and nitrogen were conducted by Atlantic Microlab, Inc., Norcross, Georgia. Gas samples were analyzed by a custom Agilent 7890B Gas Chromatograph (Agilent PorapakQ column, 6 ft, 1/8 in. OD) with a dual detector system (TCD and FID). Calibration curves for the observed gases were prepared from commercial standards of known concentration obtained from buycalgas.com.

### Electrochemical Measurements

Cyclic voltammetry was performed with a typical three-electrode setup using a CH Instruments 600E Series potentiostat. The electrochemical cell was equipped with a glassy carbon disk working electrode (3 mm diameter), a platinum wire counter electrode, and a silver wire quasi-reference electrode. Acetonitrile or DMF solutions containing 0.1 M Bu_4_NPF_6_ as the supporting electrolyte were used in all studies as specified. Ferrocene was added at the end of experiments and served as an internal standard to reference the potential. Electrolysis solutions and electrochemical cells were thoroughly degassed with nitrogen or carbon dioxide for 10–20 min prior to each experiment. Freshly made solutions were used for each experiment. Controlled potential electrolyses (CPE) were conducted in an airtight two-compartment cell with a glassy carbon rod working electrode (type 2, Alfa Aesar, 2 mm diameter), a silver wire quasi-reference electrode, and a high-surface area platinum mesh counter electrode. The platinum counter electrode was positioned inside a small isolation chamber with a fine frit. The isolation chamber contained the same solution, but without catalyst. The electrolysis solution in the working electrode compartment was continuously stirred during the experiments. Evolved gases were quantified by taking aliquots (0.30 mL) from the headspace using a sealable gastight syringe for injection into the gas chromatograph for analysis.

### X-Ray Crystallography

A single crystal coated with Paratone-N hydrocarbon oil was mounted on the tip of a MiTeGen micromount. The temperature was maintained at 200 K with an Oxford Cryostream 700 during data collection at the University of Mississippi, Department of Chemistry and Biochemistry, X-ray Crystallography Facility. Samples were irradiated with Mo-Kα radiation with λ = 0.71073 Å using a Bruker Smart APEX II diffractometer equipped with a fine-focus sealed tube source and APEX-II detector. The Bruker APEX2 v. 2009.1 software package was used to integrate raw data which were corrected for Lorentz and polarization effects (APEX2, 2009). A semi-empirical absorption correction (SADABS) was applied (Sheldrick, 2000). The space group was identified based on systematic absences, E-statistics, and successive refinement of the structure. The structure was solved using direct methods and refined by least-squares refinement on F2 and standard difference Fourier techniques using SHELXL (Sheldrick, 1990, 2008, 2014). Thermal parameters for all non-hydrogen atoms were refined anisotropically, and hydrogen atoms were included at ideal positions. Crystallographic data in CIF format was deposited into The Cambridge Crystallographic Data Center (CCDC); see deposition number CCDC 1909986 (${\text{NiL}}_{2}^{2+}$) and Table S1.

### Synthesis of the Ligand

Ligand precursor 8-bromo-2-(pyridin-2′-yl)quinoline (**2**) was synthesized as previously reported (Mao et al., 2005; Wickramasinghe et al., 2015). New ligand (8-(diphenylphosphaneyl)-2-(pyridin-2′-yl)quinoline) was prepared using a modified procedure (Murata and Buchwald, 2004; Zhang et al., 2017).

#### 8-(diphenylphosphaneyl)-2-(pyridin-2′-yl)quinoline, *L*

Inside the glovebox, to an oven-dried pressure flask with a Teflon screw cap was added 8-bromo-2-(pyridin-2′-yl)quinoline, **2** (0.80 g, 2.81 mmol), Pd(OAc)_2_ (0.032 g, 0.140 mmol), 1,1′-bis(diisopropylphosphino)ferrocene (0.071 g, 0.168 mmol), and NaO^t^Bu (0.324 g, 3.37 mmol). Then 5 mL anhydrous toluene was added and the mixture was stirred for 1 h. Diphenylphosphine (0.41 mL, 2.34 mmol) was added to the flask via a micropipette and the flask was re-sealed and heated at 120°C for 36 h. After cooling to room temperature, the reaction mixture was concentrated and purified by a deactivated silica column eluting with hexanes:ethyl acetate (5:1). The product was obtained as a light yellow solid, which is air stable in the solid phase (0.667 g, 73 %). ^1^H NMR (CD_3_CN, 500 MHz): δ 8.64 (dq, *J* = 0.7 Hz, *J* = 4.8 Hz, 1H), 8.54 (d, *J* = 8.6 Hz, 1H), 8.40 (d, *J* = 8.6 Hz, 1H), 8.04 (d, *J* = 8.0 Hz, 1H), 7.95 (d, *J* = 8.2 Hz, 1H), 7.75 (td, *J* = 1.7 Hz, *J* = 7.8 Hz, 1H), 7.50 (td, *J* = 0.8 Hz, *J* = 8.0 Hz, 1H), 7.43–7.34 (m, 11H), 7.13 (qd, *J* = 1.3 Hz, *J* = 3.8 Hz, 1H). ^13^C{^1^H} NMR (CDCl_3_, 126 MHz): δ 156.18 (s), 154.94 (s), 148.86 (s), 148.63 (d), 139.26 (d), 137.62 (d), 137.13 (s), 136.98 (s), 134.54 (s), 134.38 (s), 134.06 (s), 128.74 (s), 128.56 - 128.43 (m), 127.93 (s), 126.96 (s), 124.11 (s), 122.37 (s), 119.00 (s). ^31^P{^1^H} NMR (CDCl_3_, 167 MHz) δ−13.35. HR-ESI-MS (M^+^) m/z calc. for [L+Cs^+^]^+^, 523.0340, Found, 523.0327.

### Synthesis of the Metal Complexes

#### Common Synthetic Procedure

An acetonitrile or methanol solution (5 mL) of the NNP ligand **L** (0.050 g, 0.128 mmol) was prepared in a two-neck round bottom flask equipped with a reflux condenser before 0.5 equivalents of the corresponding metal precursor was added to the solution. The reaction mixture was refluxed for 6 h under nitrogen. After cooling to room temperature, the solvent was evaporated under reduced pressure and the residue was washed with Et_2_O.

#### ${\text{FeL}}_{2}^{2+}$, *[FeL_2_](OTf)_2_*

The complex was prepared from Fe(OTf)_2_ (0.023 g, 0.064 mmol) and 2 equivalents of **L** (0.050 g, 0.128 mmol) in acetonitrile. A short size-exclusion column (Sephadex^®^ LH-20) was run, eluting with methanol, and a single dark band was collected and dried under vacuum. The complex was further purified by crystallization from a concentrated solution of methanol by slow diffusion of diethyl ether. Yield = 0.065 g (90%). ^1^H NMR (CD_3_CN, 500 MHz): δ 9.04 (d, *J* = 8.7 Hz, 2H). 8.79 (d, *J* = 8.0 Hz, 2H), 8.62 (d, *J* = 8.7 Hz, 2H), 8.33 (br t, 2H), 8.25 (t, *J* = 7.6 Hz, 2H), 8.14 (d, *J* = 8.0 Hz, 2H), 7.65 (t, *J* = 7.65 Hz, 2H), 7.11–7.06 (m, 6H), 6.91 (t, *J* = 6.35 Hz, 2H), 6.80 (t, *J* = 7.35 Hz, 4H), 6.67 (t, *J* = 7.3 Hz, 4H), 6.31 (br t, 4H), 6.11 (br t, 4H). ^13^C{^1^H} NMR (acetone-*d*_6_, 126 MHz): δ 161.36 (s), 157.83 (t), 157.55 (s), 125.27 (s), 141.26 (s), 139.20 (s), 138.62 (s), 134.92 (s), 131.79 (t), 131.49 (t), 130.97–130.89 (m), 130.56–130.48 (m), 129.77 (t), 129.51–129.38 (m), 129.22 (s), 129.03 (s), 128.87 (s), 128.71 (s), 128.54 (s), 128.44 (s), 128.29 (s), 128.12 (d), 127.97 (s), 126.30 (s), 123.42 (s), 121.81 (s), 120.87 (s). ^31^P{^1^H} NMR (CDCl_3_, 167 MHz) δ 58.94. HR-ESI-MS (M^+^) m/z calc. for [FeL_2_(OTf)]^+^, 985.1441, Found, 985.1454.

#### [FeL_2_](Br)_2_

This complex was made in analogous fashion to [FeL_2_](OTf)_2_ as detailed above using FeBr_2_ (0.014 g, 0.064 mmol). Yield = 0.058 g (91%). ^1^H NMR (DMSO-*d*_6_, 500 MHz): δ 9.29 (d, *J* = 8.8 Hz, 2 H), 9.03 (d, *J* = 8.85 Hz, 2H), 8.95 (d, *J* = 8.1 Hz, 2H), 8.54 (d, *J* = 8.05 Hz, 2H), 8.46 (br t, 2H), 8.32 (t, *J* = 7.6 Hz, 2H), 7.79 (t, *J* = 7.8 Hz, 2H), 7.26 (d, *J* = 5.55 Hz, 2H), 7.13 (t, *J* = 7.45 Hz, 2H), 7.08 (t, *J* = 7.35 Hz, 2H), 7.02 (t, *J* = 6.25 Hz, 2H), 6.85 (t, *J* = 7.55 Hz, 4H), 6.68 (t, *J* = 7.45 Hz, 4H), 6.27 (br t, 4H), 6.03 (br t, 4H). ^13^C{^1^H} NMR (DMSO-*d*_6_, 126 MHz): δ 160.19 (s), 156.43 (t), 156.26 (s), 151.31 (s), 140.07 (s), 138.35 (s), 137.65 (s), 133.98 (s), 130.81 (s), 130.25 (t), 129.72–129.59 (m), 129.33 (s), 129.15 (t), 128.53 (t), 128.37 (t), 128.14 (s), 127.95 (s), 127.79 (s), 127.67–127.59 (m), 126.77 (s), 126.61 (s), 126.46 (s), 125.49 (s), 121.16 (s). ^31^P{^1^H} NMR (DMSO-*d*_6_, 167 MHz) δ 59.07. HR-ESI-MS (M^+^) m/z calc. for [FeL_2_Br]^+^, 915.1104, Found, 915.1077.

#### ${\text{CoL}}_{2}^{2+}$, *[CoL_*2*_](OTf)_*2*_]*

The complex was prepared from Co(CH_3_CN)_2_(OTf)_2_ (0.028 g, 0.064 mmol) and 2 equivalents of **L** in acetonitrile. A short size-exclusion column (Sephadex® LH-20) was run, eluting with methanol, and a single dark band was collected and dried under vacuum. Yield = 0.067 g (92%). HR-ESI-MS (M^+^) m/z calc. for [CoL_2_(OTf)]^+^, 988.1424, Found, 988.1389. The complex was fully characterized as a Co(III) complex, prepared by stirring a methanol solution of the Co(II) species overnight under air. Crystals of the Co(III) complex were grown from methanol by slow diffusion of diethyl ether. Elem. Anal. calc. for C_52_H_38_CoN_4_P_2_(CF_3_*SO*_3_)_3_**·**(H_2_O): C, 50.62; H, 3.09; N, 4.29. Found: C, 50.19; H, 3.05; N, 4.12. HR-ESI-MS (M^+^) m/z calc. for [CoL_2_(*OTf*)_2_]^+^, 1137.0944. Found, 1137.0947.

#### ${\text{NiL}}_{2}^{2+}$, *[NiL_*2*_](ClO_*4*_)_*2*_*

The complex was synthesized by reacting Ni(ClO_4_)_2_**·**6H_2_O (0.023 g, 0.064 mmol) and **L** (0.050 g, 0.128 mmol) in methanol. The complex was purified by recrystallization from hot methanol. Yield = 0.057 g (86%). Elem. Anal. calc. for C_52_H_38_Cl_2_N_4_NiO_8_P_2_**·**(H_2_O)_2_: C, 58.13; H, 3.94; N, 5.21. Found: C, 58.33; H, 3.86; N, 5.20. HR-ESI-MS (M^+^) m/z calc. for [NiL_2_(ClO_4_)]^+^, 937.1410. Found, 937.1398.

#### ${\text{CuL}}_{2}^{2+}$, *[CuL_*2*_](ClO_4_)_*2*_*

The complex was prepared from Cu(ClO_4_)_2_**·**6H_2_O (0.024 g, 0.064 mmol) and two equivalents of **L** in acetonitrile. The complex was found to be unstable in solutions and could not be fully purified. Yield = 0.061 g (92%). ESI-MS (M^+^) m/z calc. for [CuL_2_(ClO_4_)]^+^, 942.14. Found, 942.13.

#### ${\text{ZnL}}_{2}^{2+}$, *[ZnL_*2*_](OTf)_*2*_*

Treatment of Zn(OTf)_2_ (0.023 g, 0.064 mmol) with 2 equivalents of **L** in methanol gave the product as a light green powder. Yield = 0.065 g (89%). ^1^H NMR (CD_3_CN, 500 MHz): δ 9.13 (d, J = 8.75 Hz, 2H), 8.80 (d, *J* = 8.75 Hz, 2H), 8.56 (t, *J* = 8.55 Hz, 4H), 8.01–7.94 (m, 6H), 7.44 (br d, 2H), 7.22 (t, *J* = 7.35 Hz, 2H), 7.13 (t, *J* = 7.15 Hz, 2H), 6.94–6.89 (m, 10H), 6.70 (br m, 4H), 6.38 (br d, 4H). ^13^C{^1^H} NMR (acetone-*d*_6_, 126 MHz): δ 152.88 (s), 148.71 (s), 148.56 (s), 148.07 (t), 144.93 (s), 142.76 (s), 142.35 (s), 141.95 (s), 134.96 (t), 134.49 (s), 132.37 (s), 132.18 (t), 131.02 (t), 130.67 (d), 130.11 (t), 129.92 (t), 128.92 (s), 128.30 (t), 127.41 (t), 125.63 (s), 122.07 (s). ^31^P{^1^H} NMR (CDCl_3_, 167 MHz) δ −34.69. HR-ESI-MS (M^+^) m/z calc. for [ZnL_2_(OTf)]^+^, 993.1383, Found, 993.1382.

## Results and Discussion

### Synthesis of the Ligand and the Metal Complexes

**Figure 2:**
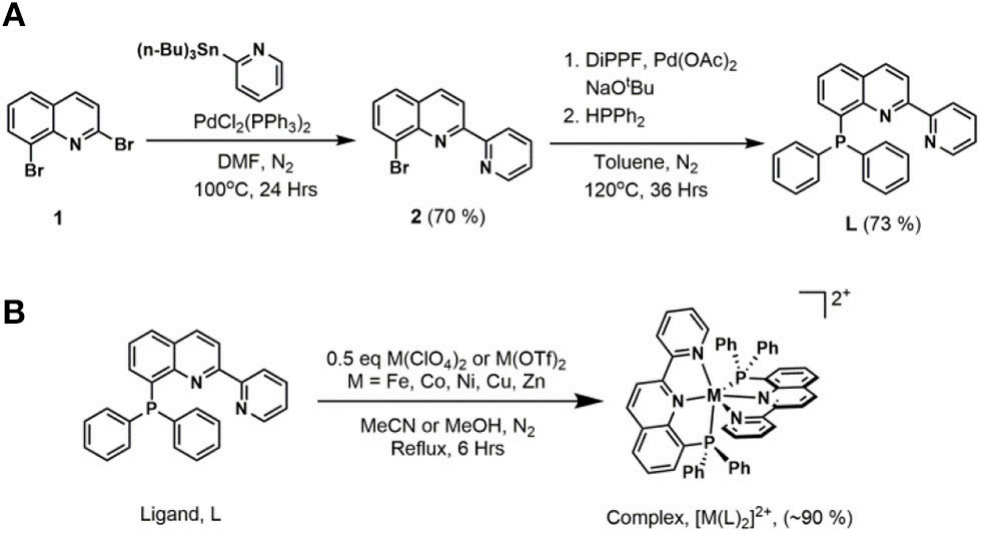
**(A)** Synthesis of 8-(diphenylphosphaneyl)-2-(pyridin-2′-yl)quinoline, ***L*. (B)** General synthetic route for the metal complexes.

Ligand precursor 2,8-dibromoquinoline **(1)** was prepared via a three-step synthetic route starting with 2-bromoaniline and cinnamoyl chloride (Wickramasinghe et al., 2015). A CombiFlash^®^ Rf+ system was utilized to obtain high purity product from the crude reaction mixture. As shown in Figure 2, Stille coupling of 2-(tributylstannyl)pyridine and **1** afforded 8-bromo-2-(pyridin-2′-yl)quinoline **(2)** (Wickramasinghe et al., 2015). The final ligand, 8-(diphenylphosphaneyl)-2-(pyridin-2′-yl)quinoline **(L)**, was prepared from **2** and diphenylphosphine using a modified cross-coupling reaction (Murata and Buchwald, 2004; Zhang et al., 2017). A deactivated silica gel column allowed purification of the product, in which 2-(pyridin-2′-yl)quinoline was found to be the only major side-product.

The metal complexes were synthesized by refluxing the appropriate metal(II) salts with 2 equivalents of the ligand **L** in acetonitrile or methanol. Metal complexes in powder form were obtained after evaporation of the solvent and washing with diethyl ether. The Fe and Co complexes were initially purified by a Sephadex^®^ column and obtained in high yields of ~90%. Further purification of all complexes was done by recrystallization from concentrated methanol or acetonitrile solutions, generally at −20°C or by slow diffusion of diethyl ether into the solution. The complexes are stable in the solid state to ambient light, air, and moisture.

In all cases, formation of the bis-chelated complexes was favored in the reaction conditions employed here. For reactions involving Co(II), Ni(II), Cu(II), and Zn(II), treatment of the appropriate metal precursor with 1 equivalent of **L** in polar solvents initially led to the formation of a mixture of mono- and bis-chelated metal complexes. Stirring the reaction mixture for prolonged periods led to clean formation of the bis-chelated complexes. In the case of Fe(II), the reaction of **L** and Fe(OTf)_2_ in a 1:1 ratio immediately formed bis-chelated complex as the only product. A similar result was observed when FeBr_2_ was used instead of Fe(OTf)_2_. This observation with iron complexes is analogous to the [(Ph-PNN)_2_Fe]^2+^ complexes reported by Zell et al. (2013). In their studies, DFT calculations indicated that formation of the bis-chelated complexes is thermodynamically favored relative to the mono-chelated compounds in all conditions. They could tune this coordination behavior by altering the substituents of the phosphine donor where bulky tert-butyl groups allowed formation of the mono-chelated complex only and isopropyl substituents gave access to both the mono- and bis-chelated complexes depending on the initial ratio of metal precursor and ligand during metalation. Basu et al. (2018) followed the same principle to avoid bis-chelation in their report.

Synthesis of the mono-chelated Co(II) complex was attempted by treating a tetrahydrofuran solution of CoCl_2_ with 1 equivalent of **L**. A green precipitate appeared immediately which was identified by mass spectroscopy to be mono-chelated **L**-CoCl_2_ complex. However, due to limited solubility in traditional non-coordinating solvents, it could not be further characterized. Dissolving the complex in polar solvents such as acetonitrile, methanol, or DMF led to a gradual color change to a reddish-brown solution, indicating formation of the bis-chelated ${\text{CoL}}_{\text{2}}^{\text{2+}}$ complex. This behavior is similar to the observation made by Harris et al. (1969) for Co(tpy) complexes.

Stirring 2:1 mixtures of the ligand and the appropriate metal precursors in methanol or acetonitrile for ~12 h led to quantitative formation of the bis-chelated complexes, ${\text{ML}}_{2}^{2+}$. Elevated temperatures significantly reduced the reaction time. Each of the ${\text{ML}}_{2}^{2+}$ complexes are stable in the solid state, and apart from the Cu(II) complex, they are also stable in solution. ${\text{CuL}}_{\text{2}}^{\text{2+}}$ slowly decomposes in solution and could not be purified or fully characterized. We note that the cobalt(II) complex is easily oxidized to the cobalt(III) complex in solution when exposed to air.

### Characterization of Metal Complexes

Elemental analyses and mass spectrometry of each ${\text{ML}}_{2}^{2+}$ complex show the 2:1 ratio of ligand to metal. The Co(II), Ni(II), and Cu(II) complexes are paramagnetic. As expected of a d^10^ metal center, the Zn(II) complex is diamagnetic. The diamagnetic nature of the d^6^ Fe(II) complex indicates a low-spin octahedral electronic state. Two distinct sets of ^1^H signals were observed for the phenyl groups in these complexes, which suggests that no plane of symmetry passes through the metal center. Notably, only one peak is observed in the ^31^P NMR spectra of ${\text{FeL}}_{2}^{2+}$ and ${\text{ZnL}}_{2}^{2+}$ signifying that the phosphorus atoms are chemically equivalent, consistent with κ^3^ coordination modes for both NNP pincer ligands and octahedral complexes.

**Figure 3:**
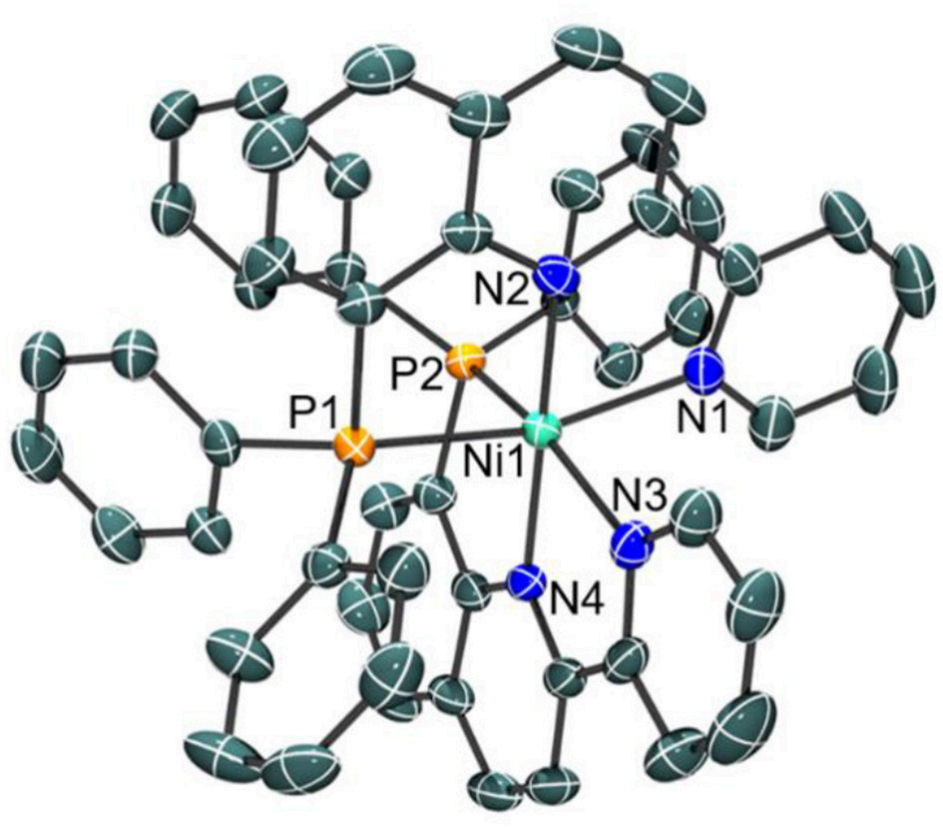
Crystal structure of the cation of **[NiL**_**2**_**](ClO**_**4**_**)**_**2**_ with thermal ellipsoids shown at the 35% probability level. Hydrogen atoms have been omitted for clarity. Selected bond distances: Ni–N(1), 2.086(4); Ni–N(2), 2.062(4); Ni–N(3), 2.093(4); Ni–N(4), 2.054(4); Ni–P(1), 2.4323(13); Ni–P(2), 2.4183(13) Å.

Indeed, a crystal structure of the octahedral nickel complex was obtained as shown in Figure 3. Single crystals of ${\text{NiL}}_{2}^{2+}$ were grown from a concentrated solution of hot methanol that was allowed to cool to room temperature. The nickel-nitrogen bond distances range from 2.054(4) to 2.093(4) Å, with pyridine donors *trans* to the phosphine donor having an average Ni-N bond distance of 2.090 Å while those that are *trans* to another pyridine have an average Ni-N bond distance of 2.058 Å. Significantly longer bond distances are observed for the phosphine donors at 2.4323(13) for Ni-P(1) and 2.4183(13) for Ni-P(2) in the solid state.

For the three reported paramagnetic complexes, solution magnetic susceptibilities (μ_eff_) were determined by the Evans method (Evans, 1959). At room temperature, the values are 2.6, 2.8, and 2.0 for ${\text{CoL}}_{2}^{2+}$, ${\text{NiL}}_{2}^{2+}$, and ${\text{CuL}}_{2}^{2+}$, respectively. The experimental values are close to previously reported values of similar octahedral complexes and characteristic of 1, 2, and 1 unpaired electron(s) for the Co(II), Ni(II), and Cu(II) complexes, respectively. The relatively high μ_eff_ value for cobalt (2.6) is typical of cobalt polypyridyl complexes, and indicates significant orbital contribution (Chen et al., 2018).

### Electrochemistry

**Figure 4:**
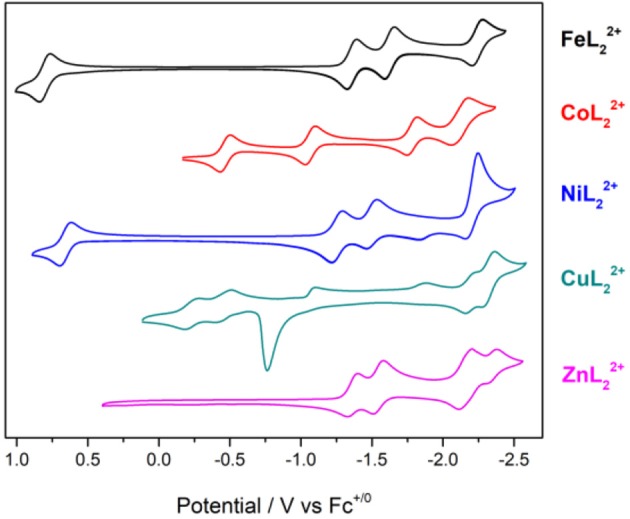
Cyclic voltammograms of [ML_2_]^2+^ complexes of Fe, Co, Ni, Cu, and Zn at 1 mM concentrations in CH_3_CN/0.1 M Bu_4_NPF_6_ under N_2_. Scan rate: 100 mV/s; glassy carbon disk working electrode.

Cyclic voltammetry was performed on the series of metal complexes to assess and compare the redox potentials in acetonitrile/0.1 M Bu_4_NPF_6_ solutions under inert atmosphere. The cyclic voltammograms are shown in Figure 4 and *E*_1/2_ values (peak potentials for the irreversible redox features) are summarized in Table S2. All potentials are reported against the ferrocenium/ferrocene couple (V vs. Fc^+/0^).

The cyclic voltammogram (CV) of ${\text{ZnL}}_{\text{2}}^{\text{2+}}$ shows four redox couples at −1.36, −1.54, −2.16, and −2.35 V. Since this complex has a redox-inactive metal center, these processes are assigned to ligand-based redox events. The zinc complex is helpful in identifying metal- and ligand-based redox processes in the remaining complexes. Notably, ${\text{ZnL}}_{\text{2}}^{\text{2+}}$ is unstable under repeated scans. The redox couples at −2.16 and −2.35 V become irreversible and a visible change in peak intensities is observed presumably due to passivation of the working electrode.

The reduction waves at *E*_*p, c*_ = −2.22 and −2.36 V of ${\text{CuL}}_{2}^{2+}$ can be safely assigned to ligand-based reductions. A sharp irreversible oxidation peak at −0.76 V is also observed, indicative of adsorption on the electrode surface. The redox features of the copper complex also lose their reversibility and peak shape under repeated scans.

Reversible metal-based M^III/II^ redox couples occur at *E*_1/2_ = 0.80, −0.46, and 0.66 V for ${\text{FeL}}_{\text{2}}^{\text{2+}}$, ${\text{CoL}}_{\text{2}}^{\text{2+}}$, and ${\text{NiL}}_{\text{2}}^{\text{2+}}$, respectively. In addition, three reversible redox events are observed at negative potentials with the iron and cobalt complexes in the potential range studied here. The nickel complex shows slightly different behavior with two reversible reductions at −1.28 and −1.53 V, and a quasireversible reduction at −2.25 V. The quasireversible reduction has two small return oxidations at −2.15 and −1.83 V. The most negative features of these complexes are assigned to ligand-based reductions by comparison to the zinc analog.

### Electrochemical Reduction of CO_2_

Next, the complexes were analyzed by cyclic voltammetry to probe their reactivity toward CO_2_ reduction. We focused on ${\text{FeL}}_{2}^{2+}$, ${\text{CoL}}_{2}^{2+}$, and ${\text{NiL}}_{2}^{2+}$ as the copper and zinc complexes were not stable under electrochemical reduction. DMF solutions containing 0.1 M Bu_4_NPF_6_ were employed for electrocatalytic studies for two reasons. First, using acetonitrile resulted in a significant amount of precipitation during controlled potential electrolysis (CPE) experiments. This is consistent with previously reported observations with 3d transition metal terpyridine complexes and ruthenium polypyridyl complexes. It was concluded that the build-up of ${\text{HCO}}_{3}^{-}$/${\text{CO}}_{3}^{2-}$ during bulk electrolyses resulted in precipitation of the complexes as ${\text{HCO}}_{3}^{-}$ or ${\text{CO}}_{3}^{2-}$ salts (Chen et al., 2011; Elgrishi et al., 2014). We confirmed the formation of carbonate in CPE experiments with ${\text{CoL}}_{2}^{2+}$ by barium triflate titration (Yang et al., 2018), consistent with the reductive disproportionation of CO_2_ to CO and ${\text{CO}}_{3}^{2-}$. The use of DMF solved this solubility issue as no precipitate was observed during electrolyses over extended periods of time. Second, using DMF gave us the opportunity to directly compare the performance of these complexes with the previously reported terpyridine systems (Elgrishi et al., 2014, 2015).

Under N_2_ atmosphere, CVs of ${\text{FeL}}_{2}^{2+}$, ${\text{CoL}}_{2}^{2+}$, ${\text{NiL}}_{2}^{2+}$, and ${\text{ZnL}}_{2}^{2+}$ in DMF (shown in Figure S1) exhibit similar behavior to that in acetonitrile, but with significantly lower stability for the nickel and zinc compounds. Redox potentials of the complexes in DMF are summarized in Table 1. Comparing the CV of the cobalt complex to its zinc counterpart, we assign the redox couples (*E*_1/2_) at −1.06 and −1.74 V to a metal-based Co^II/I^ process and to a ligand-based process, respectively. The open circuit potential of the ${\text{CoL}}_{2}^{2+}$ was measured at −0.61 V, which is consistent with these assignments. A reversible ligand-based wave was also observed at *E*_1/2_ = −2.07 V. Adding free ligand to the solution increases the intensity of this peak (Figure S2), which further supports the assignment of this feature as a ligand-based reduction. Scanning past this ligand-based reduction gives rise to an irreversible ligand-based reduction at −2.65 V, which was found to be unstable under repeated scans (Figure S3).

**Table 1:** Reductive peak potentials and diffusion coefficients (*D*) of 0.5 mM solutions of ${\text{FeL}}_{2}^{2+}$, ${\text{CoL}}_{2}^{2+}$, ${\text{NiL}}_{2}^{2+}$, and ${\text{ZnL}}_{2}^{2+}$ in DMF/0.1 M Bu_4_NPF_6_ under N_2_.

**Complex**	***E_***p*1, *c***_***	***E_***p*2, *c***_***	***E_***p*3, *c***_***	***E_***p*4, *c***_***	***D* (cm^**2**^ s^**−1**^)**
${\text{FeL}}_{2}^{2+}$	−1.43	−1.67	−2.31	–	4.69 × 10^−6^
${\text{CoL}}_{2}^{2+}$	−1.09	−1.77	−2.12	−2.65	1.02 × 10^−6^
${\text{NiL}}_{2}^{2+}$	−1.32	−1.51	−1.83	−2.25	4.36 × 10^−6^
${\text{ZnL}}_{2}^{2+}$	−1.61	−1.83	−2.24	−2.41	–

*Glassy carbon working electrode, platinum wire counter electrode, and silver wire quasi-reference electrode*.

For ${\text{FeL}}_{2}^{2+}$, reversible waves with *E*_1/2_ values at −1.40, −1.64, and −2.27 V are attributed to ligand-based reductions by comparison with ${\text{ZnL}}_{2}^{2+}$. Different behavior was observed with ${\text{NiL}}_{2}^{2+}$ relative to the Fe and Co complexes with one reversible redox feature at *E*_1/2_ = −1.27 V, followed by one irreversible reduction at *E*_*p, c*_ = −1.51 V and two quasireversible reductions at *E*_*p, c*_ = −1.83 and −2.25 V. The open circuit potential was determined to be −0.68 V. Thus, the reversible redox process at −1.27 V is expected to be a metal-centered Ni^II/I^ couple, while the remaining waves are consistent with ligand-based redox events. Irreversible reductions are presumably due to dissociation of one of the tridentate ligands from the complex. This redox behavior is different from the related Co(tpy)${}_{2}^{2+}$ and Ni(tpy)${}_{2}^{2+}$ systems (Hamacher et al., 2009; Elgrishi et al., 2014). CVs of ${\text{FeL}}_{2}^{2+}$, ${\text{CoL}}_{2}^{2+}$, and ${\text{NiL}}_{2}^{2+}$ at scan rates ranging from 10 to 1,000 mVs^−1^ show that the peak currents change linearly with the square root of the scan rate (Figures S5–S7), consistent with diffusion-controlled homogeneous species. The diffusion coefficients were calculated from the slopes of the linear fits of the first reduction peaks using the Randles-Sevcik equation (Bard and Faulkner, 2001) and are presented in Table 1.

**Figure 5:**
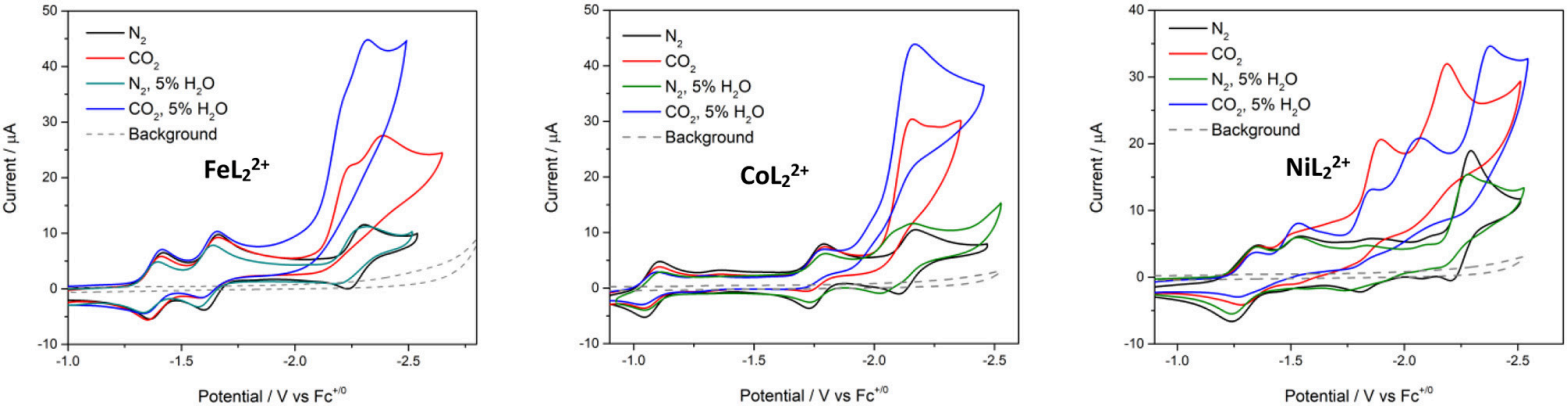
Cyclic voltammograms of ${\text{FeL}}_{2}^{2+}$, ${\text{CoL}}_{2}^{2+}$, and ${\text{NiL}}_{2}^{2+}$ at 0.5 mM concentrations in DMF/0.1 M Bu_4_NPF_6_ under N_2_ (black), CO_2_ (red), N_2_ with 5% H_2_O (green), and CO_2_ with 5% H_2_O (blue). Scan rate = 100 mV/s; glassy carbon disk working electrode.

Water was added as a proton source to investigate the proton-coupled reduction of CO_2_ with these complexes. The concentration of added water was kept fixed at 5% in these studies. No significant current enhancement was observed in the CVs when water was introduced to the systems under N_2_ (Figure 5). Likewise, there was little change to the CV of ${\text{CoL}}_{\text{2}}^{\text{2+}}$ with added H_2_O. The third (most negative) reduction peak of ${\text{FeL}}_{2}^{2+}$ and ${\text{NiL}}_{2}^{2+}$ becomes irreversible in the presence of H_2_O.

In CO_2_-saturated anhydrous solutions, the third reduction of all three complexes loses reversibility and shows current enhancement, suggesting catalytic reactivity toward CO_2_ (Figure 5). A 2.4-fold current increase was observed at the third reduction peak of ${\text{FeL}}_{2}^{2+}$ with the appearance of a new peak at *E*_*p, c*_ = −2.23 V. The new peak showed current enhancement of 1.9-fold. In ${\text{CoL}}_{2}^{2+}$, the most negative reduction peak was catalytically enhanced with an *i*_*cat*_*/i*_*p*_ value of 2.9 (where *i*_*cat*_ is the limiting catalytic current obtained under CO_2_ and *i*_*p*_ is the reductive peak current of the catalyst in the absence of substrate). CVs of ${\text{NiL}}_{2}^{2+}$ showed current enhancement of 3.5- and 1.7-fold in the third and fourth reduction waves, respectively.

Additional current enhancement at the terminal reduction peaks of the ${\text{FeL}}_{2}^{2+}$ and ${\text{CoL}}_{2}^{2+}$ systems was observed when 5% water was added to CO_2_-saturated solutions, indicating that the complexes are capable of catalyzing the proton–coupled reduction of CO_2_ (Figure S10). The CV of ${\text{NiL}}_{2}^{2+}$ under the same conditions showed a decrease in the initial catalytic waves, but an additional and larger catalytic wave appeared at *E*_*p, c*_ = −2.40 V (Figure 5).

To better understand the reactivity of the complexes and to quantify the products, a series of controlled potential electrolyses (CPEs) were performed at different applied potentials with and without added H_2_O. Gaseous products were analyzed by periodic sampling of the headspace of the airtight electrochemical cell. Table 2 summarizes the product distribution and Faradaic efficiencies (FE) of each complex over the first 2 h. Representative charge vs. time plots are provided in Figures S11–S13, S16. Carbon monoxide and hydrogen were the major products detected in all cases. A trace amount of methane was also found in the electrolyses of ${\text{CoL}}_{2}^{2+}$ and ${\text{FeL}}_{2}^{2+}$ but corresponds to <1% of the total charge passed.

**Table 2:** Summary of 2 h CPEs with ${\text{FeL}}_{2}^{2+}$, ${\text{CoL}}_{2}^{2+}$, and ${\text{NiL}}_{2}^{2+}$ (0.5 mM catalyst concentration) in CO_2_-saturated DMF/0.1 Bu_4_NPF_6_ solutions (glassy carbon rod).

**Complex**	**H^**+**^ Source**	***E_***appl***_* (V)**	**Charge (C)**	**Total FE (%)**	**FE_**CO**_ (%)**	**FE_**H2**_ (%)**
${\text{CoL}}_{2}^{2+}$	5% H_2_O	−2.01	2.07	33	16	17
	5% H_2_O	−2.06	2.94	30	23	7
	5% H_2_O	−2.11	5.51	58	16	42
	5% H_2_O	−2.16	6.77	50	11	39
	None	−2.11	2.92	6	6	–
	5% MeOH	−2.12	5.56	41	7	34
${\text{NiL}}_{2}^{2+}$	5% H_2_O	−2.06	1.18	5	5	–
	5% H_2_O	−2.28	1.37	8	8	–
${\text{FeL}}_{2}^{2+}$	5% H_2_O	−2.15	5.21	27	1	26
	5% H_2_O	−2.25	5.67	26	< 1	25
	None	−2.23	1.53	< 1	< 1	–
${\text{ZnL}}_{2}^{2+}$	5% H_2_O	−2.20	0.81	< 1	< 1	–

*For the CPE with ${\text{ZnL}}_{\text{2}}^{\text{2+}}$, a 2 mM concentration of the complex was used. All values are the average of at least two runs*.

**Figure 6:**
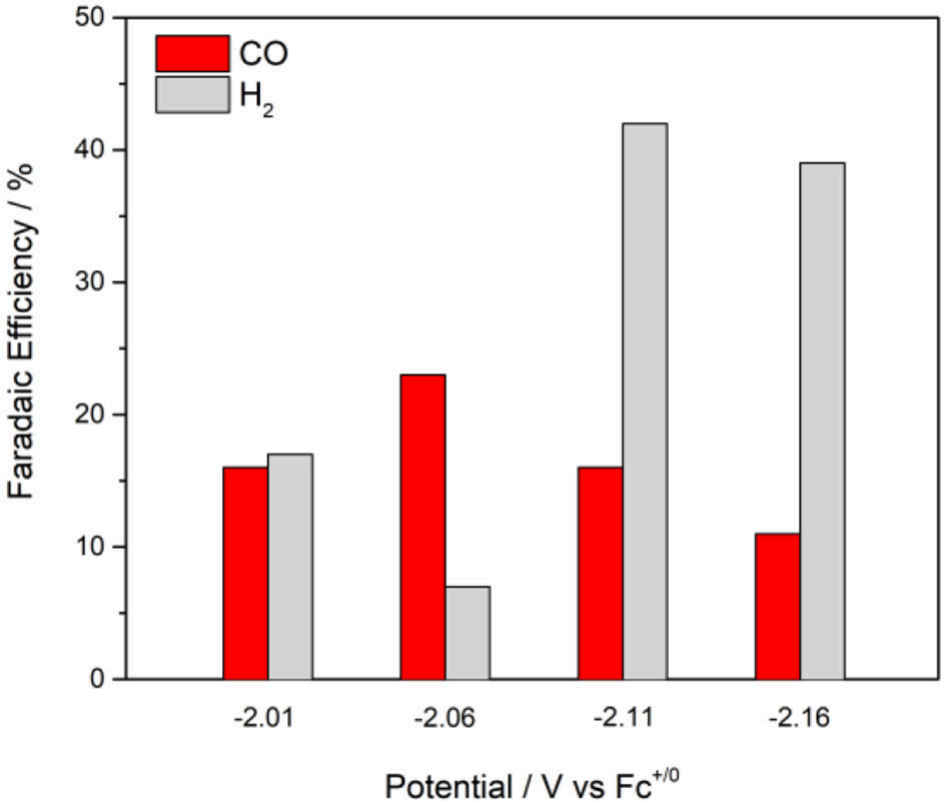
Faradaic efficiencies and product distributions from control potential electrolyses at different applied potentials with 0.5 mM ${\text{CoL}}_{2}^{2+}$ in CO_2_-saturated DMF/0.1 M Bu_4_NPF_6_ solutions containing 5% H_2_O, glassy carbon rod.

The maximum overall FE was found to be 58% for ${\text{CoL}}_{2}^{2+}$ at an applied potential of −2.11 V in the presence of 5% water (Figure 6). A maximum FE of 23% for evolved CO was recorded at *E*_*appl*_ = −2.06 V. The production of H_2_ varied with the applied potential from 7 to 42%. When methanol—a more acidic proton source—was used, the overall FE decreased, but the ratio for H_2_ production increased (Table 2). No evolved H_2_ was observed when electrolyses were performed in anhydrous solutions. However, Faradaic efficiencies for CO were lower. Rinse tests were performed on the glassy carbon rod after CPEs to investigate possible adsorption or deposition of a heterogeneous catalyst on the electrode surface. There was no visible deposition and the glassy carbon rod did not exhibit any catalytic activity in fresh CO_2_-saturated DMF/0.1 M Bu_4_NPF_6_ solution, which suggests catalysis is governed by a molecular cobalt species.

The turnover frequency (TOF) of ${\text{CoL}}_{2}^{2+}$ was calculated as previously described (Narayanan et al., 2016; Yang et al., 2018) from scan rate-dependent linear sweep voltammograms performed in N_2_- and CO_2_-saturated solutions containing 5% H_2_O. A plot of the *i*_*cat*_/*i*_*p*_ current ratio at different scan rates as a function of potential is shown in Figure S8. Although a scan rate-independent TOF was not completely reached at scan rates up to 1 V/s, an estimated TOF of 12.4 s^−1^ was determined (Figure S9).

CPEs of the ${\text{NiL}}_{2}^{2+}$ complex show that the complex is selective for CO_2_ reduction over proton reduction, albeit with very low FEs. The maximum FE observed with the nickel complex was 8%. The total charge passed was also very low compared to its iron and cobalt counterparts. The ${\text{FeL}}_{2}^{2+}$ complex primarily catalyzed proton reduction in the presence of H_2_O. Indeed, the FE for CO production was ~1% in anhydrous DMF solutions or with 5% added H_2_O. Under anhydrous conditions, proton reduction was suppressed, but CO production remained low. Rinse tests were also performed following electrolyses of both the nickel and iron complexes. For ${\text{FeL}}_{2}^{2+}$, the electrode did not show any sign of deposition. On the other hand, visible signs of deposition on the glassy carbon rod were observed with ${\text{NiL}}_{2}^{2+}$ and the electrode showed catalytic activity in fresh solutions that signals decomposition of the complex occurs to form an active heterogeneous material.

## Discussion

In general, to be an effective catalyst for CO_2_ or H^+^ reduction, a metal complex should be able to activate the substrate molecule via an open coordination site at the metal center. Thus, molecular catalysts are typically designed with at least one open or labile coordination site (Chiericato et al., 2000; Benson et al., 2009; Thoi et al., 2013; Su et al., 2018). In contrast, the complexes investigated here form an active catalyst *in situ* by ligand loss or dissociation of a donor moiety during electrolysis. Given the rigidity of the ligand, we hypothesize that one of the ligands completely dissociates. Thus, the bis-chelated ${\text{ML}}_{2}^{2+}$ complex acts as a precatalyst, which under reducing conditions loses a ligand to generate a mono-chelated, catalytically active species (bearing a single pincer ligand) with available coordination sites for substrate activation and conversion. Our attempts to isolate the mono-chelated active species were unsuccessful. However, controlled potential electrolysis of ${\text{CoL}}_{2}^{2+}$ was performed under inert atmosphere at an applied voltage of −2.65 V, and the electrolysis solution was analyzed by mass spectrometry, which showed evidence of free ligand that was not present in pre-electrolysis solutions (Figure S4). In addition, CVs taken before and after electrolysis of ${\text{CoL}}_{2}^{2+}$ show the formation of new redox features, indicating that a new electroactive species is formed under reducing conditions (Figure S14). Together, these experiments indicate that a mono-chelated complex is formed when the pre-catalyst is subjected to electrochemical reduction, and it is this species that is responsible for the catalytic activity observed in the presence of substrates (CO_2_ and H^+^).

Given the structural similarities, ${\text{CoL}}_{2}^{2+}$ likely operates by the mechanism proposed by Elgrishi et al. (2014) and Elgrishi et al. (2015) for Co(tpy)${}_{2}^{2+}$ complexes. The low Faradaic efficiencies are also consistent with their findings, which showed that the reduced mono-chelated active species can dimerize to give a deactivated species that does not reduce CO_2_. Moreover, the redox-active ligand liberated during creation of the active catalyst can be reduced further, contributing to the low overall Faradaic efficiencies (Table 2). Proposed mechanisms for CO_2_ reduction as well as the competing hydrogen evolution reaction are shown in Figure S18 for the cobalt(II) complex.

The ${\text{FeL}}_{2}^{2+}$ and ${\text{NiL}}_{2}^{2+}$ complexes behave differently than the Fe and Ni bis(terpyridine) complexes reported by Elgrishi et al. (2014). In their studies, ${\text{Fe}(\text{tpy})}_{2}^{2+}$ did not show any current enhancement in CO_2_-saturated solutions containing a proton source. Conversely, the ${\text{Ni}(\text{tpy})}_{2}^{2+}$ complex showed considerable catalytic activity under comparable conditions to those employed here and selectively produced CO in bulk electrolyses. The redox potentials of the bis(tpy) complexes and the present systems are also significantly different under N_2_ atmosphere. It was concluded from CVs of ${\text{Fe}(\text{tpy})}_{2}^{2+}$, ${\text{Ni}(\text{tpy})}_{2}^{2+}$, and ${\text{Zn}(\text{tpy})}_{2}^{2+}$ that they undergo ligand-based reductions exclusively in the potential window studied. The current enhancement observed in CVs of the complexes studied here under anhydrous conditions may be a result of CO_2_ binding to the reduced active species to form a stable intermediate, which is not effective for catalysis and does not liberate a reduced carbon product. Related observations have been reported for molecular nickel complexes that have poor Faradaic efficiencies for CO_2_ reduction despite encouraging results from cyclic voltammetry (Narayanan et al., 2016; Lieske et al., 2018). The irreversible reductions and dissimilarity between the anodic and cathodic scans of ${\text{NiL}}_{2}^{2+}$ under N_2_ indicates formation of a new species. Consistent with the rinse test, ligand dissociation likely occurs from the nickel complex during electrolysis, which is followed by formation of a heterogeneous material on the electrode surface. Decomposition of the molecular species following reduction may account for the low Faradaic efficiencies.

${\text{ZnL}}_{2}^{2+}$ was electrochemically and spectroscopically analyzed to better understand the underlying mechanism and stability of the catalysts. The zinc complex did not show any notable current increase in CO_2_-saturated solutions by cyclic voltammetry (Figure S15). It also performed poorly in CPEs with an overall FE of <1%. However, the diamagnetic nature of ${\text{ZnL}}_{2}^{2+}$ was used to obtain insight into the decomposition pathway of the reported complexes. We conducted an electrochemical experiment as described by Elgrishi et al. (2014) and Fuchs et al. (1981), in which ${\text{ZnL}}_{2}^{2+}$ was subjected to bulk electrolysis and the electrolyzed product was studied by ^1^H NMR. NMRs were taken of the solution before and after electrolysis, and after adding iodomethane as an alkylating agent (Figure S17). Free ligand peaks were visible in the ^1^H NMR spectrum of the electrolyzed solution after a 3-h CPE, consistent with ligand dissociation. Changes in the aromatic region of the NMR spectrum and appearance of new peaks in the aliphatic region after alkylation suggest possible dearomatization and carboxylation of the ligand (Fuchs et al., 1981; Elgrishi et al., 2014).

While our cobalt system suffers from low overall Faradaic efficiency and poor selectivity, the ligand structure is amenable to electronic and steric modifications. Elgrishi et al. (2015) were able to tune the selectivity of ${\text{Co}(\text{tpy})}_{2}^{2+}$ complexes by introducing different substituents on the tpy ligand. They improved selectivity toward CO production by disfavoring the hydrogen evolution reaction (HER), but overall Faradaic efficiencies remained low. The best derivative featuring bulky tert-butyl substituents on the tpy ligand led to the highest overall FE at 41%, presumably by reducing catalyst deactivation via dimerization. However, these results indicate that ligand modifications are largely ineffective for improving catalyst stability and thus they were not pursued with the NNP ligand investigated here.

An early example of electrochemical CO_2_ reduction was reported by Arana et al. (1992) involving bis-chelated Fe, Co, and Ni complexes of redox-active NNN-type ligands. These complexes showed CO_2_ reduction activity to various extents based on their current response in cyclic voltammetry studies. Among them, ${\text{Co}(\text{dapa})}_{2}^{2+}$ and ${\text{Ni}(\text{dapa})}_{2}^{2+}$ complexes (dapa = 2,6-bis-[1-(phenylimino)ethyl]pyridine) showed the best activity in CVs. Under inert atmosphere, both complexes showed one metal-based and two ligand-based reductions, similar to the complexes reported here. Catalytic current was observed at the second reduction for these systems, whereas catalysis begins in earnest at the third reduction with our system. However, the stability and substrate selectivity of the catalysts were not reported. Only a limited product analysis was done for the cobalt system, which had a ~60% FE for formic acid production. This clearly suggests that this system follows a different catalytic mechanism than ours and the Fontecave systems where CO was the sole carbon-containing product.

As discussed previously, introducing steric bulk in pincer ligands can enforce mono-chelation, thereby avoiding the ligand dissociation step needed to access available coordination sites for substrate binding to the metal core. In addition, they can obstruct dimerization of reduced intermediates. Recently, electrocatalytic CO_2_ reduction with cobalt and nickel complexes featuring modified dapa ligands were reported by Kang and Rochford, respectively. Mono-chelation was achieved by introducing diisopropyl groups in the ligand framework (Narayanan et al., 2016; Liu et al., 2018). The Co complex showed minimal reactivity toward CO_2_ and was not studied further, whereas the Ni complex showed a good current response in CVs under CO_2_. However, during bulk electrolysis, the Ni complex produced only a small amount of CO with nearly quantitative formation of H_2_. Liu et al. (2018) hypothesized that catalysis could be improved by replacing one imino group with a pyridyl moiety. Their new NNN ligand has a redox-active bipyridine unit with extended π-conjugation and a sterically bulky imino group on one side. With this ligand, the corresponding cobalt complex shows metal- and ligand-based redox events under argon. Adding water to the system gave rise to new waves corresponding to a Co-hydride species, which was found to mediate formate production in CO_2_-saturated solution. Consistent with DFT calculations, the reduced species in the presence of substrate can access two different pathways to generate both CO and formate, where formate is produced via a Co^II^-H intermediate. Despite similarities between this ligand and the NNP ligand reported here, our ${\text{CoL}}_{2}^{2+}$ complex has different product selectivity and CO was found to be the only 2e^−^ reduced C_1_ product. This is likely due to a difference in hydricity of the Co^II^-H intermediate formed in our case, in which CO_2_ insertion into the metal-hydride to make a formato intermediate is not competitive with protonation to form H_2_ (Thoi et al., 2013; Kang et al., 2015).

Examples of phosphine-based pincer-ligated 3d metal complexes are still scarce among CO_2_ reduction electrocatalysts. Recently, a manganese tri-carbonyl complex with a symmetric PNP-pincer ligand was reported by the Richeson group. This complex shows an unusual coordination environment with a *mer*-M(CO)_3_ motif rather than the usual *fac*-M(CO)_3_ configuration (Rao et al., 2016). Moreover, this complex also triggers CO_2_ reduction at its first reduction. Addition of H_2_O as a weak proton source increased the current and lowered the catalytic overpotential, but also reduced substrate selectivity as HER activity was enhanced.

Pincer ligands with *N*-heterocylic carbene (NHC) donors have also been employed in CO_2_ reduction. A nickel-based catalyst for CO_2_ reduction supported by a mixed donor CNC-type ligand was reported by Sheng et al. (2015). This distorted square planner complex has a labile acetonitrile ligand at the fourth position and shows three irreversible reductions under N_2_. Among them, the first reduction at −1.19 V (vs Fc^+/0^) was assigned to the Ni^II/I^ couple. Catalysis occurs at the most negative reduction in CO_2_-saturated CH_3_CN. Added water was found to enhance catalysis without compromising product selectivity. Myren et al. (2019) recently reported two manganese tricarbonyl complexes with similar CNC pincer ligands which can reduce CO_2_ to CO at the first reduction potential. Though the overpotential is large, these catalysts also exhibit high selectivity in the presence of a proton source.

Cope et al. (2017) replaced the pyridyl group of the Sun catalyst with an aryl donor to prepare a Ni complex ligated by an anionic CCC-pincer ligand. This more electron-rich ligand on nickel results in faster catalysis (30x TOF), at the expense of a higher overpotential, and produces a mixture of CO and formate. Electrocatalysis occurs at the first reduction with this catalyst and added water enhances CO_2_ reduction and the rate of catalysis. Bulk electrolysis experiments confirmed that the catalyst is selective, producing CO and formate with an optimal FE of 81% for reduced carbon products. However, catalysis slows down after ~1 h of electrolysis for unspecified reasons.

## Conclusions

A rigid NNP-type pincer ligand and its mid-to-late first-row transition metal complexes have been synthesized. The ${\text{ML}}_{2}^{2+}$ complexes reported here are the first examples of an asymmetric phosphine-substituted pincer ligand employed for electrocatalytic CO_2_ reduction. Despite the steric bulk of the phenyl substituents on this ligand, the metal ions prefer to coordinate with two ligands to form pseudo-octahedral bis-chelated complexes. Electrochemical studies confirm that the cobalt complex is capable of catalyzing the electrochemical reduction of CO_2_ to CO. However, catalytic activity is greatly limited by deleterious side reactions. We reason that the rigid NNP framework inhibits ligand dissociation from the reduced complex and, thus, impedes formation of the catalytically active species.

## Author Contributions

KT synthesized and characterized the catalysts, collected and analyzed data, and wrote the original draft. AI synthesized and characterized the catalysts and collected data. JJ conceptualized the experiments, analyzed data, supervised research activities, acquired funding, and revised the manuscript.

### Conflict of Interest Statement

The authors declare that the research was conducted in the absence of any commercial or financial relationships that could be construed as a potential conflict of interest.
