# Supplementary material for: Synthesis of a Redox-Active NNP-Type Pincer Ligand and Its Application to Electrocatalytic CO2 Reduction With First-Row Transition Metal Complexes
Source: Front Chem. 2019 May 21;7:330. doi: 10.3389/fchem.2019.00330 (PMC6536570; doi:10.3389/fchem.2019.00330)
Supplement: Supplementary file 1 [file Data_Sheet_1.pdf]

Synthesis of a redox-active NNP-type pincer ligand and its application to first-row transition metal complexes for electrocatalytic CO_2 reduction

Kallol Talukdar, Asala Issa, and Jonah W. Jurss*

Department of Chemistry and Biochemistry, University of Mississippi, University, MS 38677, USA.

***jwjurss@olemiss.edu**

-Supporting Information-

Table of Contents

NMR spectra of the ligand, ***L***.......... 3

NMR spectra of ***[FeL$_2$](OTf)$_2$***.......... 4

NMR spectra of ***[FeL$_2$]Br$_2$***.......... 6

NMR spectra of ***[ZnL$_2$](OTf)$_2$***.......... 7

Crystal data and structure refinement for **NiL$_2$$^{2+}$**.......... 9

Redox potentials of the **M(NNP)$_2$$^{2+}$** complexes in acetonitrile.......... 10

CVs of **FeL$_2$$^{2+}$**, **CoL$_2$$^{2+}$**, **NiL$_2$$^{2+}$**, and **ZnL$_2$$^{2+}$** in DMF.......... 10

CVs of Co(II) complex at different metal to ligand ratio.......... 11

Repeated CVs of **CoL$_2$$^{2+}$** under N_2 atmosphere.......... 11

HR-MS of controlled potential electrolysis solution of **CoL$_2$$^{2+}$** under N_2.......... 12

Scan rate dependence studies of **FeL$_2$$^{2+}$**, **CoL$_2$$^{2+}$**, and **NiL$_2$$^{2+}$**.......... 13

Kinetics of **CoL$_2$$^{2+}$** ($i_{cat}/i_p$ vs potential and TOF vs scan rate).......... 16

CVs of **FeL$_2$$^{2+}$**, **CoL$_2$$^{2+}$**, and **NiL$_2$$^{2+}$** under dry and wet conditions.......... 17

Representative Charge vs Time plots of CPEs.......... 18

Representative CVs before and after CPE experiment of **CoL$_2$$^{2+}$**.......... 19

CV, Charge vs Time plot, and NMR experiment of **ZnL$_2$$^{2+}$**.......... 20

Proposed mechanisms for CO_2 and H^+ reduction by **CoL$_2$$^{2+}$**.......... 22

L, **^{1}H NMR (500 MHz, CD_3CN)**

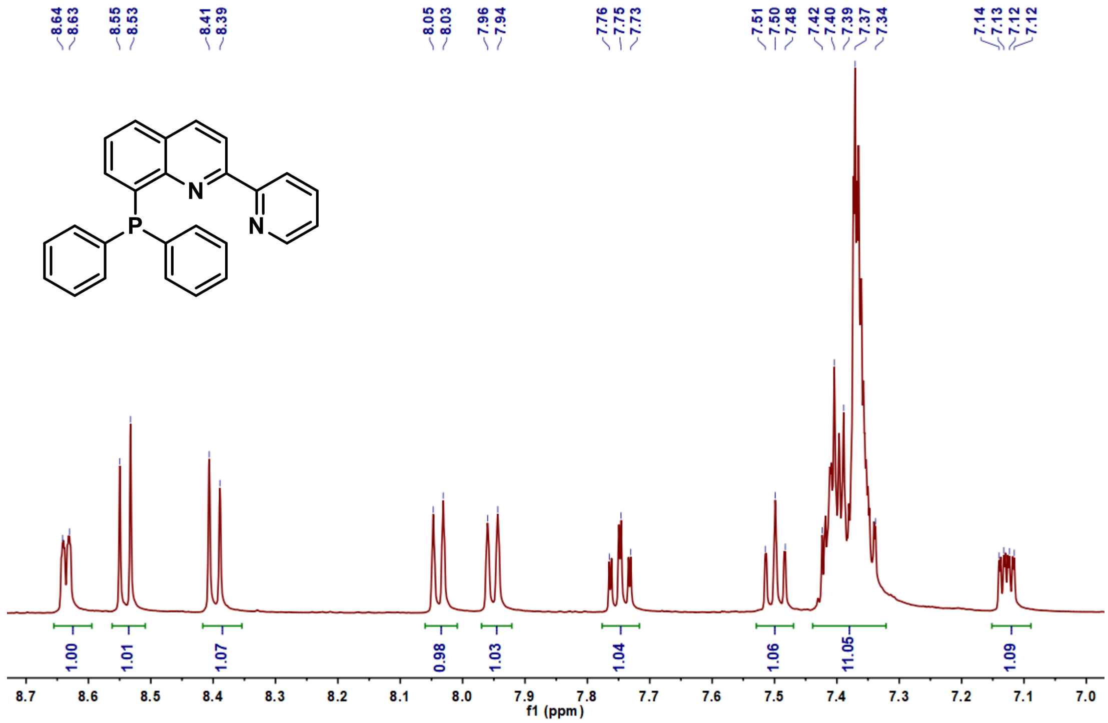

L, **^{31}P NMR (167 MHz, $CDCl_3$)**

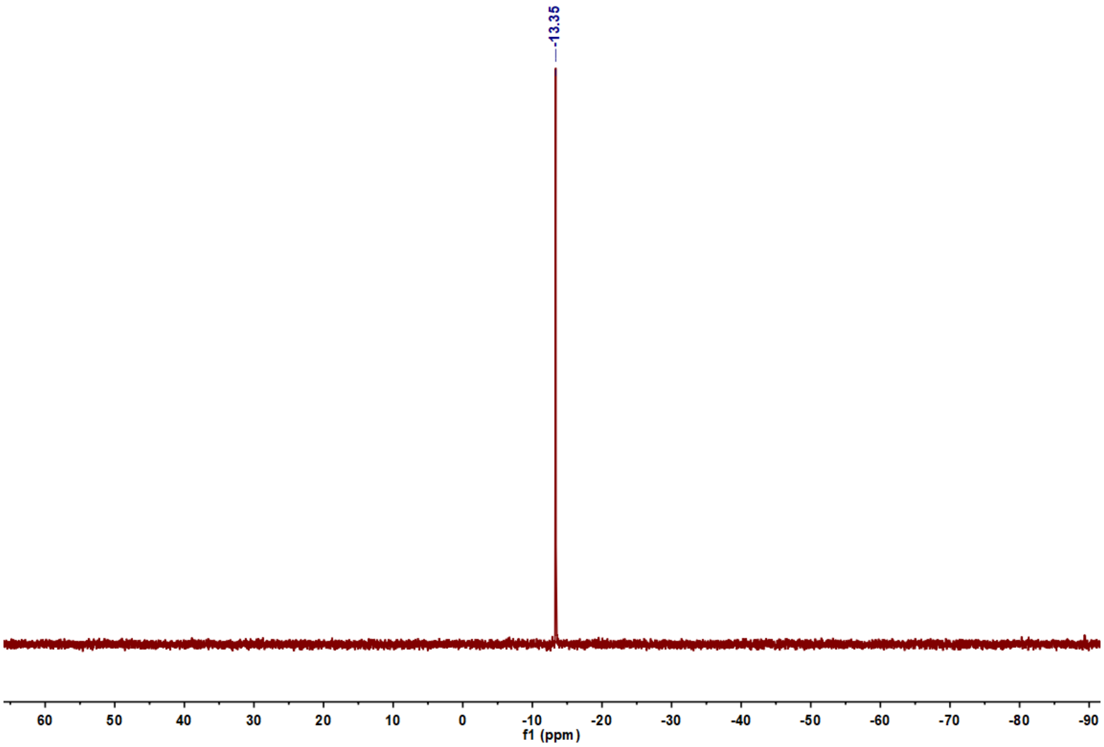

***L*, ^{13}C NMR (126 MHz, $CDCl_3$)**

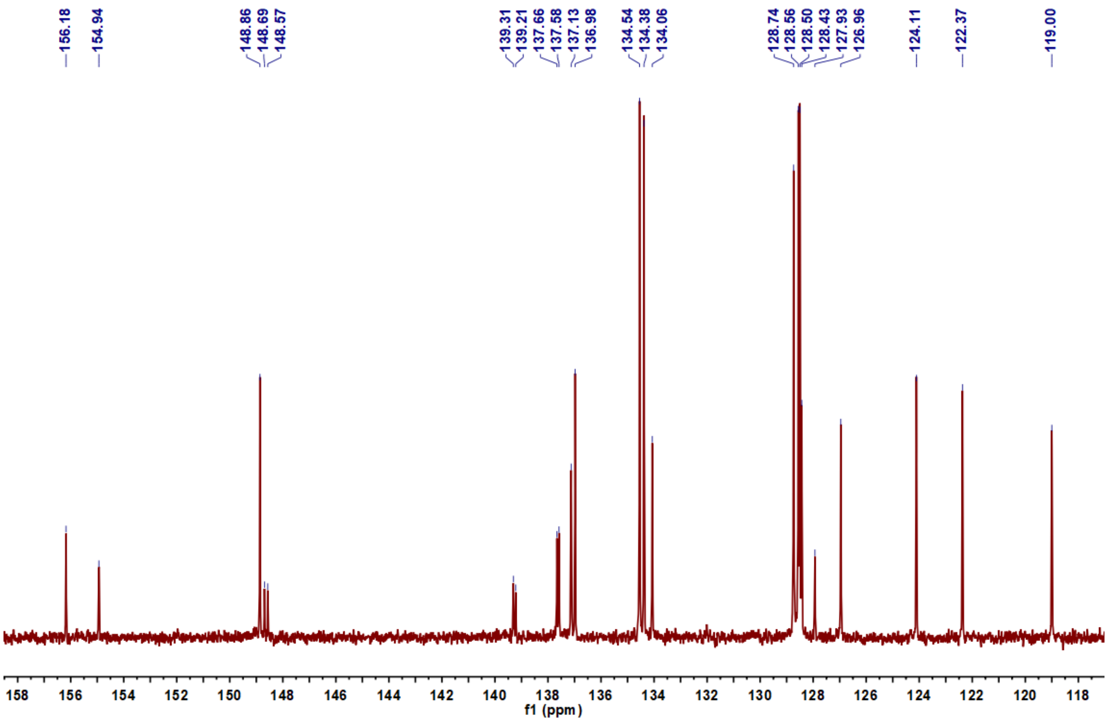

$[FeL_2](OTf)_2$, ^{1}H NMR (500 MHz, CD_3CN)

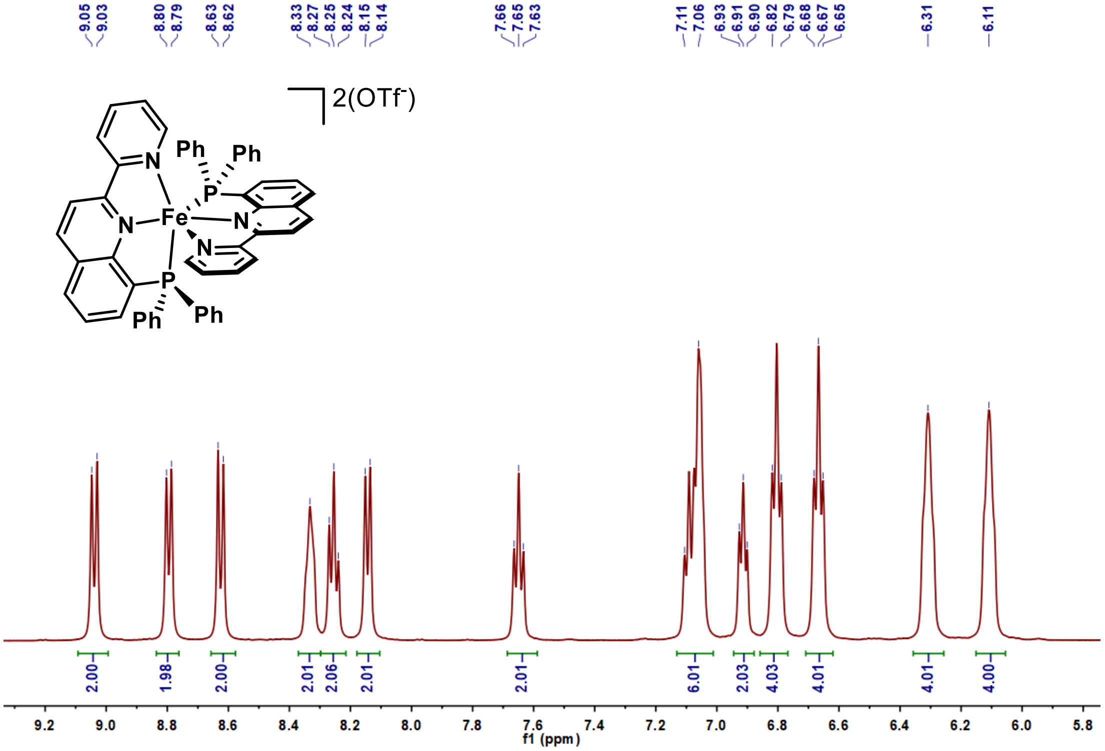

***[FeL₂](OTf)₂*, ³¹P NMR (167 MHz, CDCl₃)**

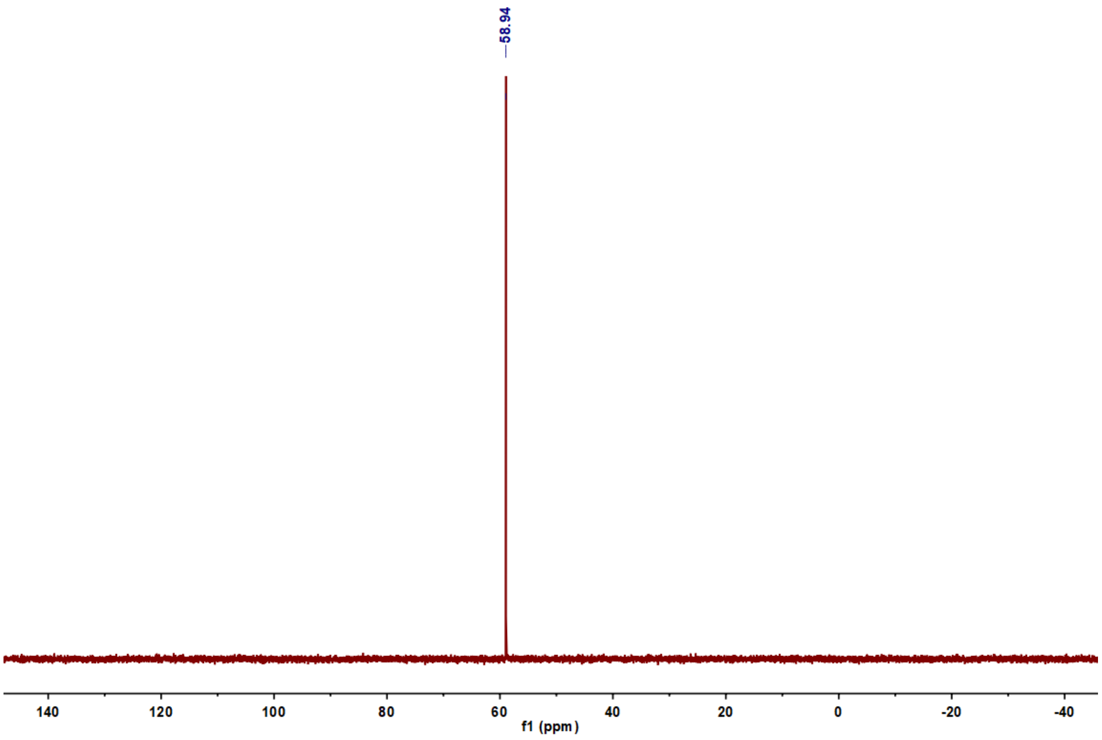

***[FeL₂](OTf)₂*, ¹³C NMR (126 MHz, Acetone-*d₆*)**

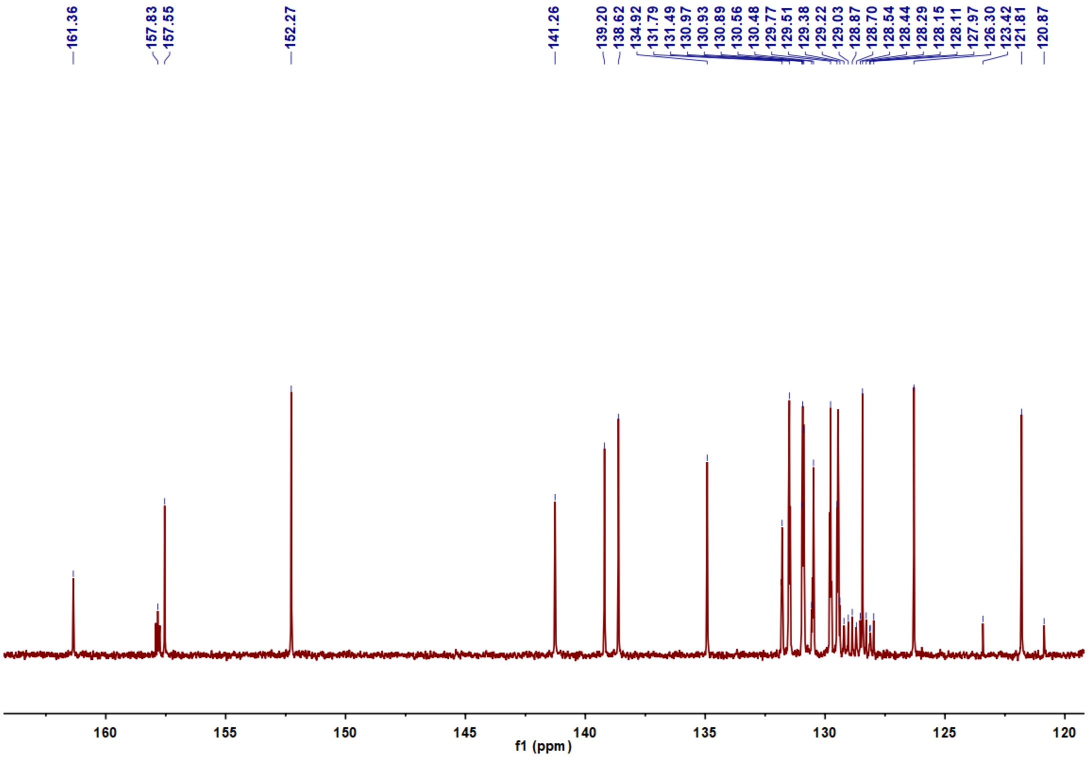

[FeL$_2$]Br$_2$, ^{1}H NMR (500 MHz, DMSO-d_6)

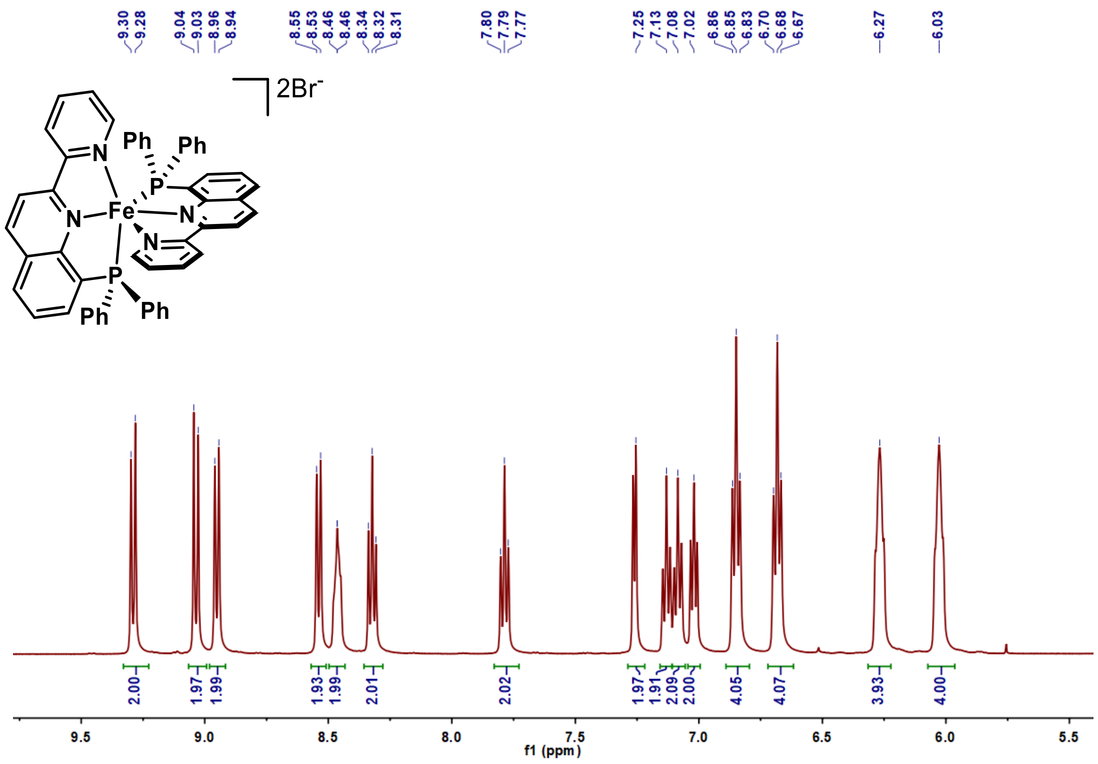

[FeL$_2$]Br$_2$, ^{31}P NMR (167 MHz, DMSO-d_6)

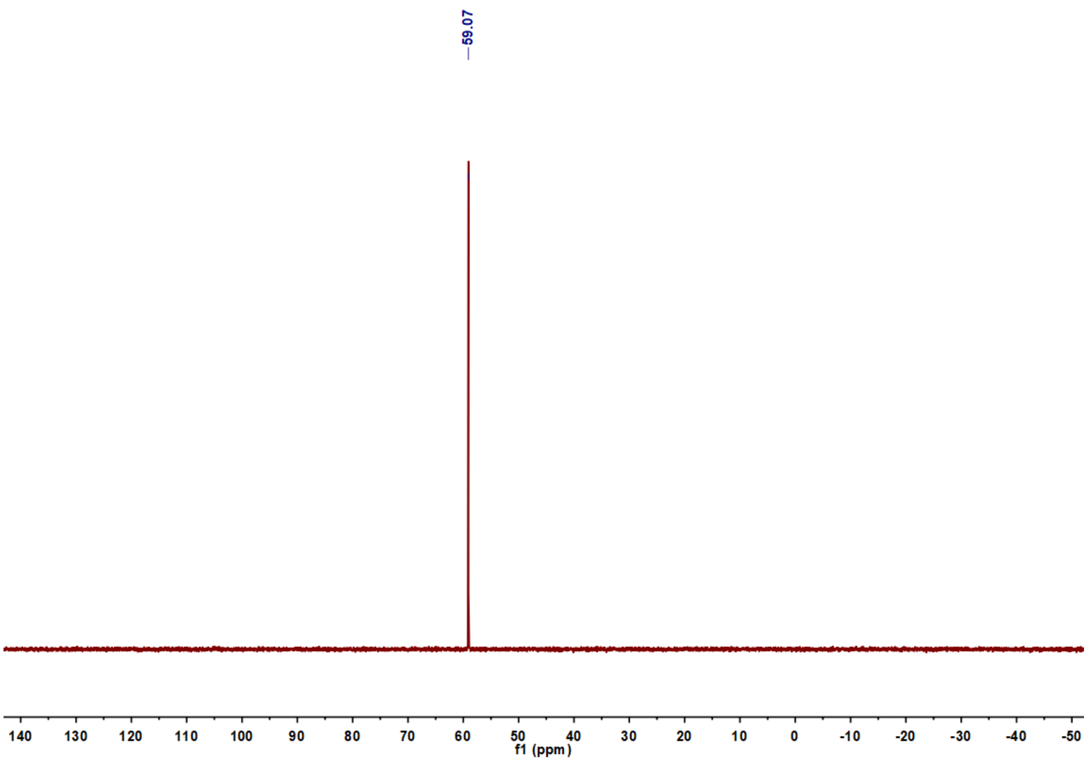

[FeL$_2$]Br$_2$, **^{13}C NMR (126 MHz, DMSO-*d$_6$*)**

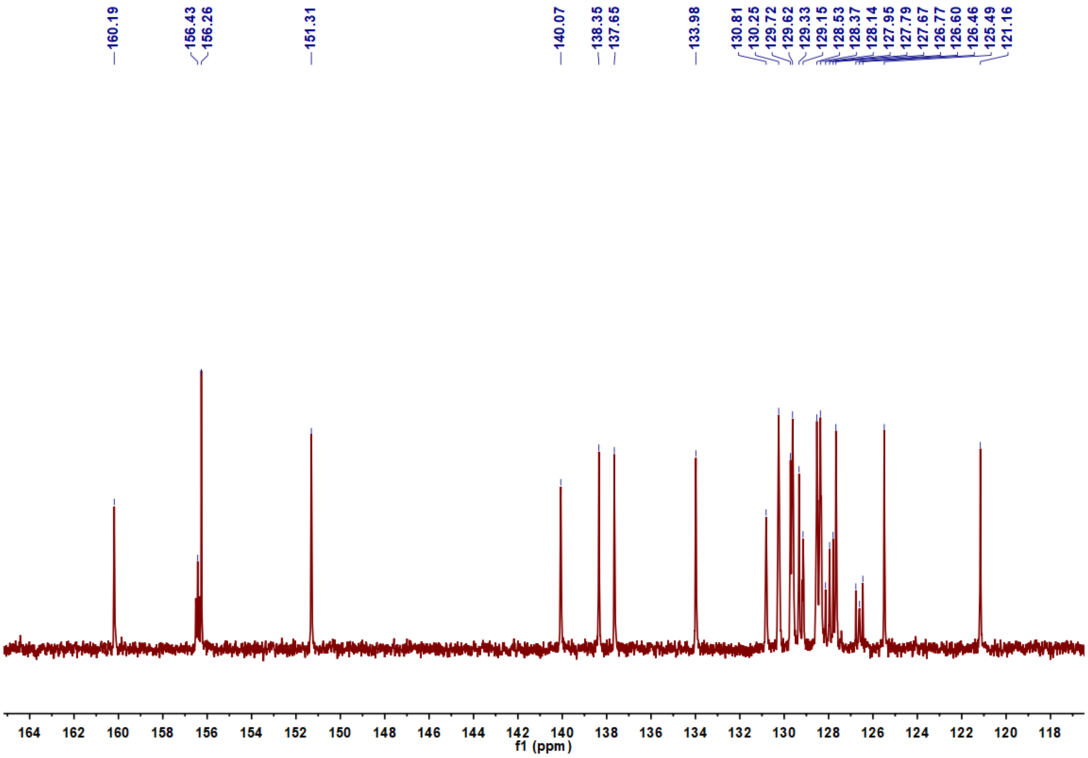

[ZnL$_2$](OTf)$_2$, **^{1}H NMR (500 MHz, CD$_3$CN)**

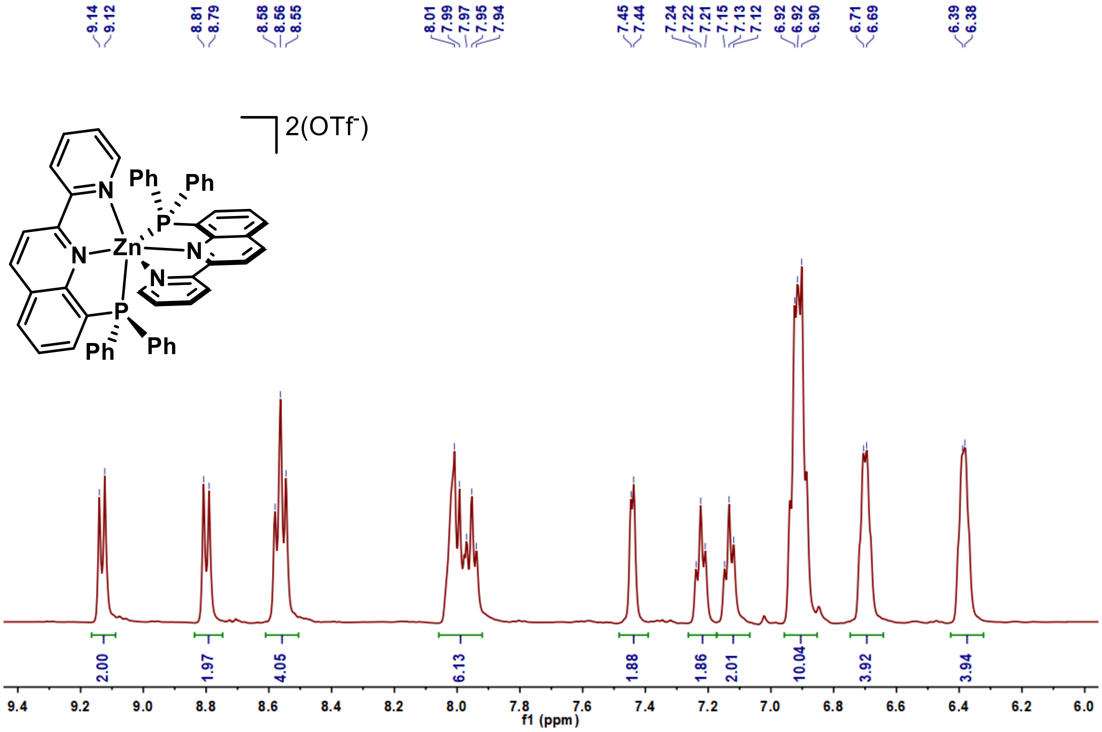

***[ZnL$_2$](OTf)$_2$*, ^{31}P NMR (167 MHz, CDCl$_3$)**

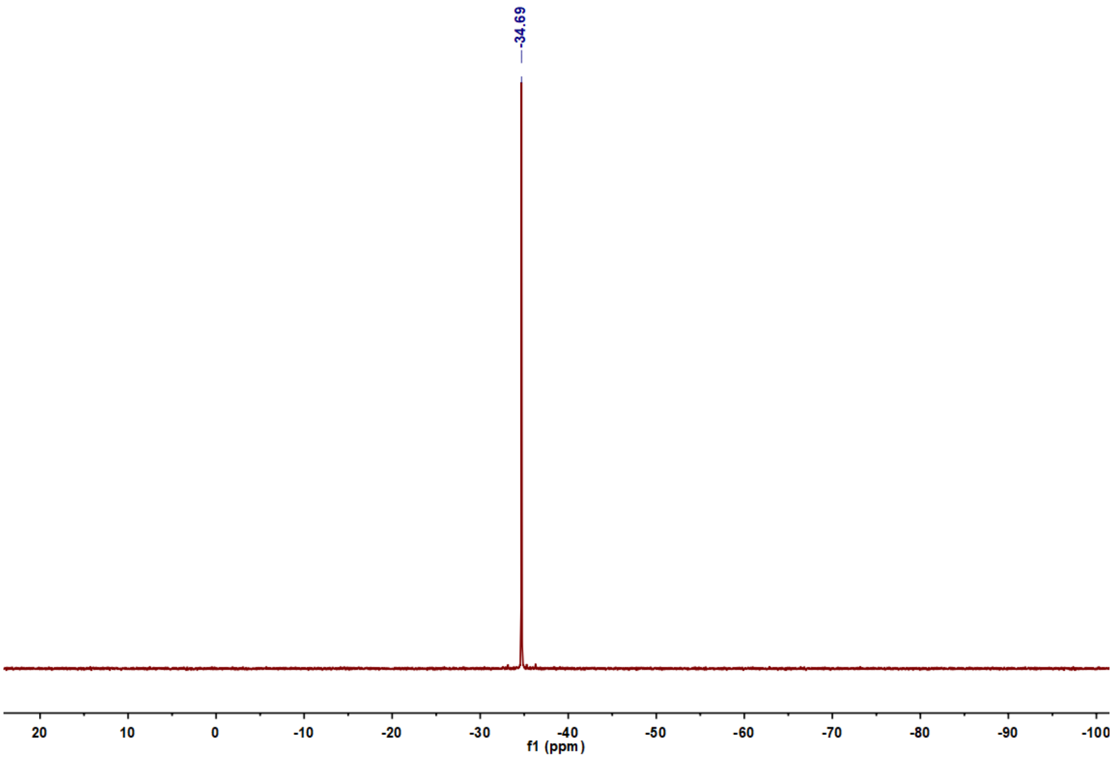

***[ZnL$_2$](OTf)$_2$*, ^{13}C NMR (126 MHz, Acetone-*d$_6$*)**

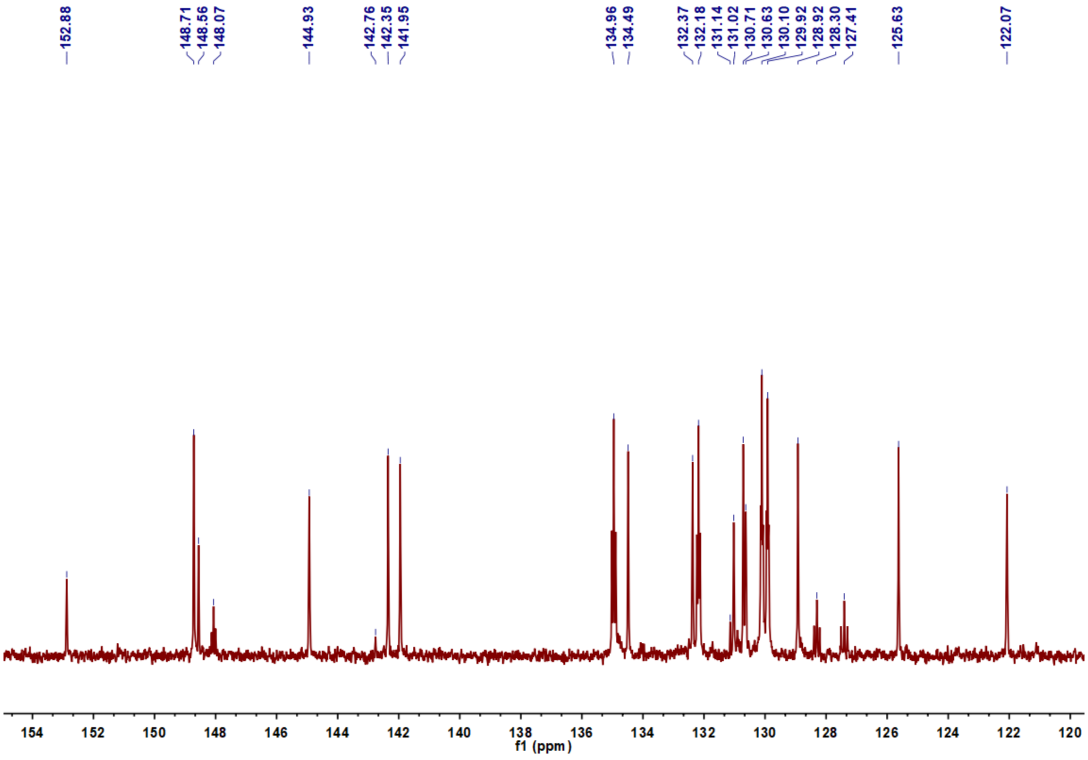

Table S1. Crystal data and structure refinement for **NiL_2^{2+}** (CCDC deposition #: 1909986).

Empirical formula	C54 H46 Cl2 N4 Ni O10 P2	
Formula weight	1102.50	
Temperature	200(2) K	
Wavelength	0.71073 Å	
Crystal system	Monoclinic	
Space group	P2(1)/n	
Unit cell dimensions	a = 11.383(3) Å	α= 90°
	b = 17.434(5) Å	β= 96.227(5)°
	c = 25.580(7) Å	γ = 90°
Volume	5047(2) Å^3	
Z	4	
Density (calculated)	1.451 Mg/m^3	
Absorption coefficient	0.618 mm^{-1}	
F(000)	2280	
Crystal size	0.20 x 0.20 x 0.20 mm^3	
Theta range for data collection	1.42 to 25.33°.	
Index ranges	-12<=h<=13, -20<=k<=20, -30<=l<=30	
Reflections collected	34960	
Independent reflections	9131 [R(int) = 0.0356]	
Completeness to theta = 25.00°	99.7 %	
Absorption correction	None	
Max. and min. transmission	0.8863 and 0.8863	
Refinement method	Full-matrix least-squares on F^2	
Data / restraints / parameters	9131 / 1 / 662	
Goodness-of-fit on F^2	1.160	
Final R indices [I>2sigma(I)]	R1 = 0.0698, wR2 = 0.1723	
R indices (all data)	R1 = 0.0768, wR2 = 0.1765	
Largest diff. peak and hole	1.031 and -0.737 e.Å^{-3}	

Electrochemical Data

Table S2. Redox properties of $M(NNP)_2^{2+}$ complexes in acetonitrile / 0.1 M Bu_4NPF_6 solutions under N_2. Scan rate = 100 mV/s, glassy carbon disk working electrode. All potentials are referenced versus the ferrocenium/ferrocene ($Fc^{+/0}$) couple.

**Complex**	**Redox Potentials, $E_{1/2}$ (V vs $Fc^{+/0}$)**						
**FeL_2^{2+}**	0.80	-1.36	-1.63	-	-2.24	-	-
**CoL_2^{2+}**	-	-0.46	-1.06	-1.78	-2.12	-	-
**NiL_2^{2+}**	0.66	-1.25	-1.50	-1.83[b]	-2.15[b]	-2.25[c]	-
**CuL_2^{2+} [a]**	-0.22	-0.46	-0.76[b]	-1.10[c]	-1.87[c]	-2.18	-2.32
**ZnL_2^{2+} [a]**	-	-1.36	-1.54	-2.16	-2.35	-	-

a. Unstable under repeated cycles; *b*. Irreversible ($E_{p,a}$); *c*. Irreversible / quasireversible ($E_{p,c}$)

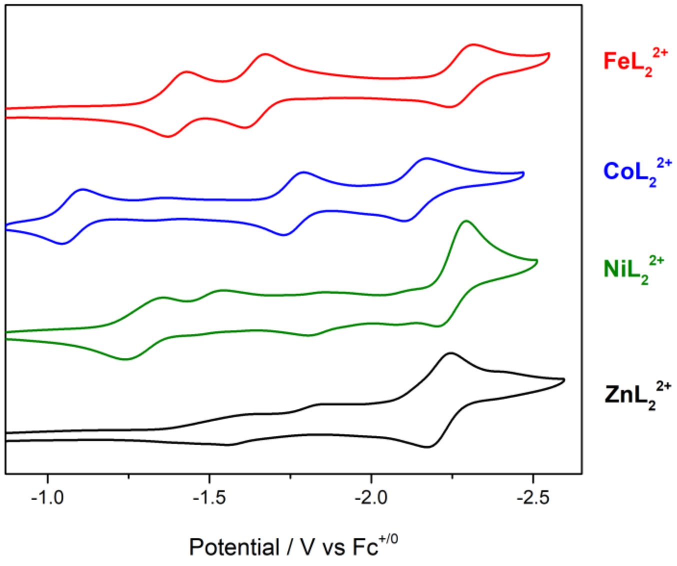

Fig. S1. Cyclic voltammograms of **FeL_2^{2+}**, **CoL_2^{2+}**, **NiL_2^{2+}**, and **ZnL_2^{2+}** at 0.5 mM concentrations in DMF / 0.1 M Bu_4NPF_6 under N_2. Scan rate = 100 mV/s; glassy carbon disk working electrode, platinum wire counter electrode, and silver wire quasi-reference electrode.

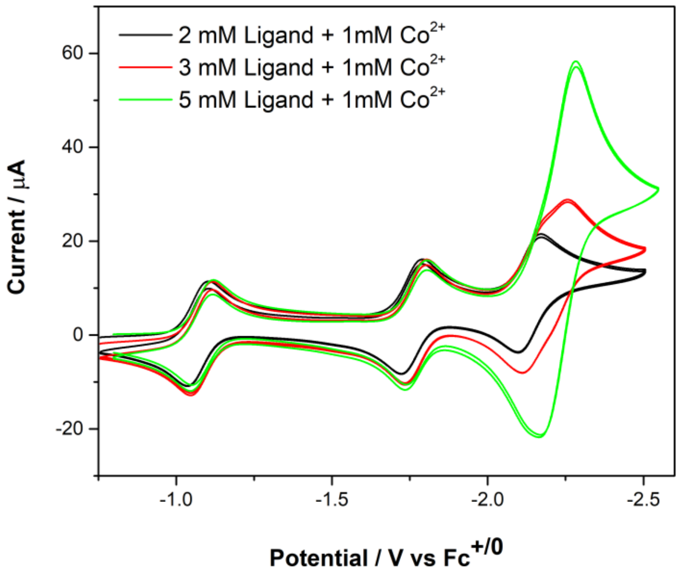

Fig. S2. Cyclic voltammograms of $\mathbf{CoL_2}^{2+}$ prepared *in situ* by adding different amounts of metal precursor and ligand in DMF / 0.1 M Bu_4NPF_6 under N_2. Scan rate = 100 mV/s; glassy carbon disk working electrode, platinum wire counter electrode, and silver wire quasi-reference electrode.

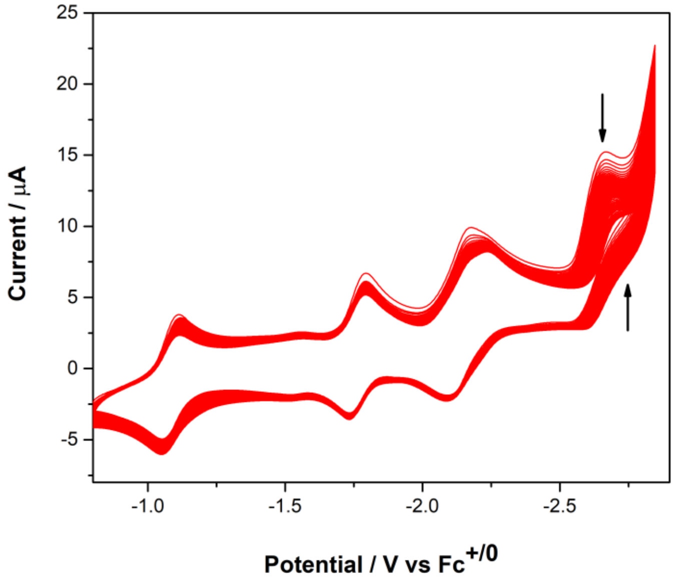

Fig. S3. Repeated cyclic voltammograms of $\mathbf{CoL_2}^{2+}$ under N_2 atmosphere in DMF / 0.1 M Bu_4NPF_6. Scan rate = 100 mV/s; glassy carbon disk working electrode, platinum wire counter electrode, and silver wire quasi-reference electrode.

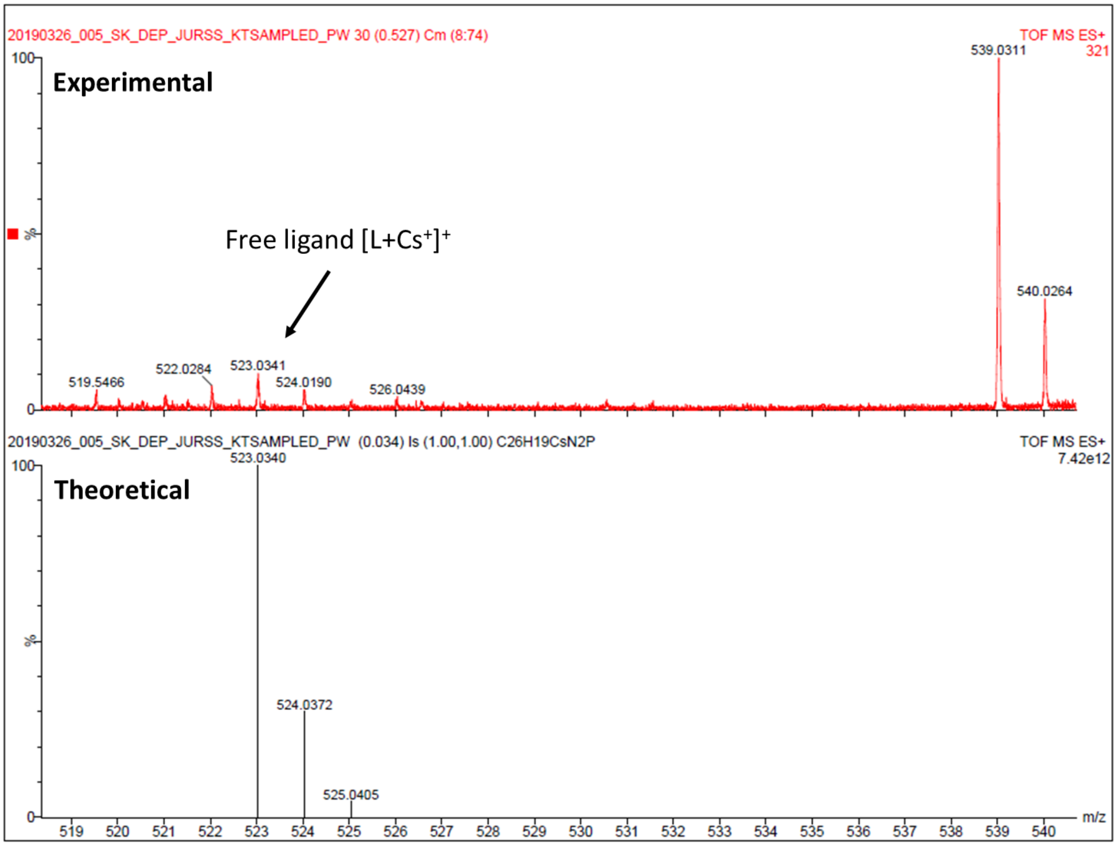

Fig. S4. High resolution mass spectrum of 2 mM $\mathbf{CoL_2^{2+}}$ in N_2-saturated DMF / 0.1 M Bu_4NPF_6 solution after controlled potential electrolysis at E_{appl} = -2.65 V. The sample was analyzed after 45 minutes of electrolysis. Glassy carbon rod working electrode, platinum wire counter electrode, and silver wire quasi-reference electrode.

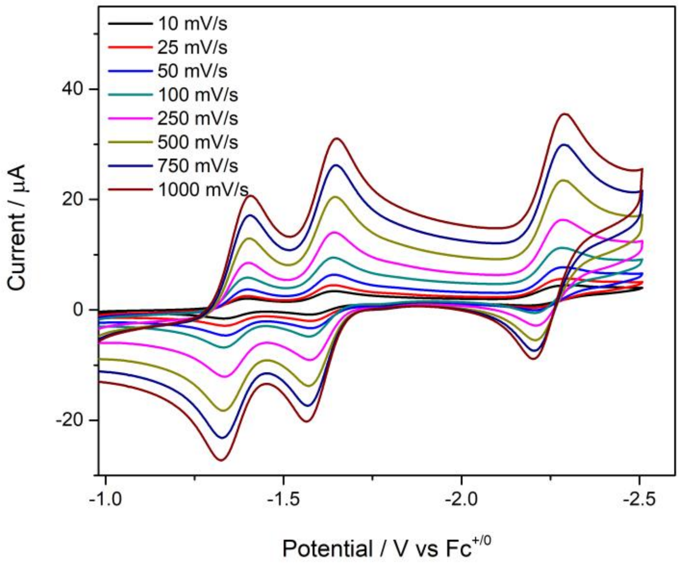

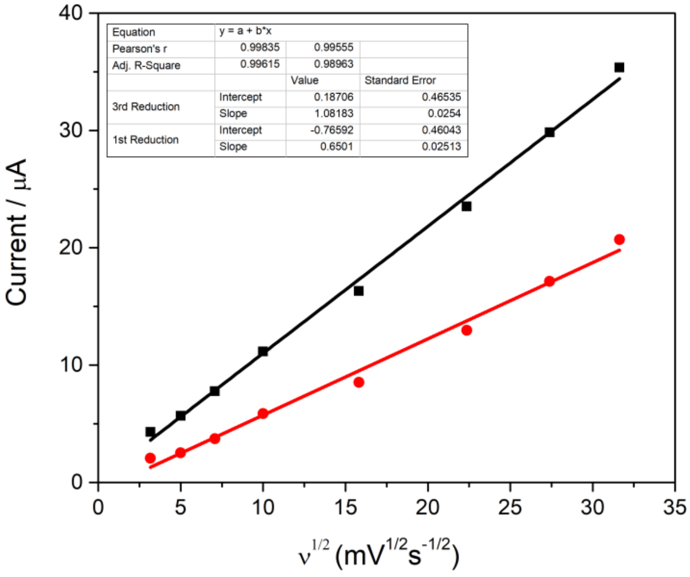

Fig. S5. (Top) CVs of 0.5 mM **FeL_2^{2+}** under N_2 atmosphere at different scan rates in DMF / 0.1 M Bu_4NPF_6. Glassy carbon disk working electrode, platinum wire counter electrode, and silver wire quasi-reference electrode.

(Bottom) Plot of reductive peak currents of the first reduction (red circles) and third reduction (black squares) from cyclic voltammograms versus the square root of the scan rate.

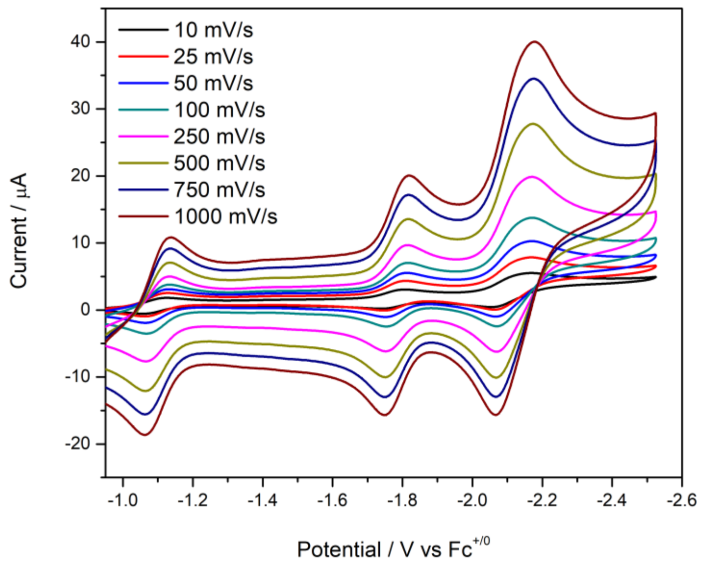

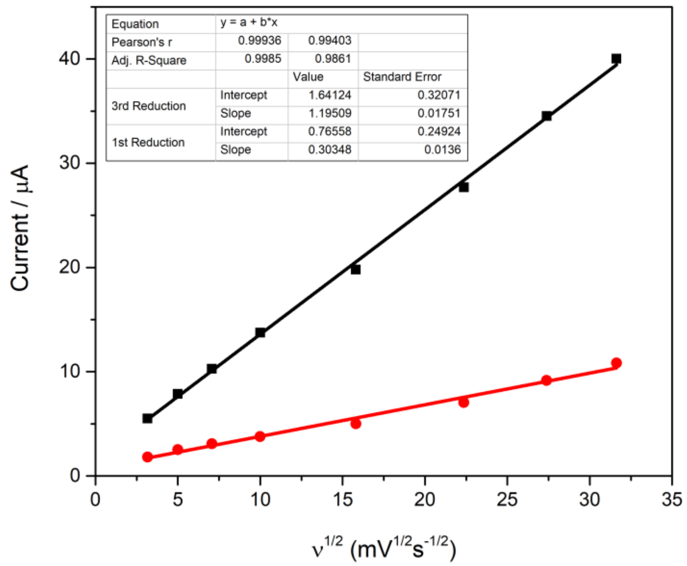

Fig. S6. (Top) CVs of 0.5 mM **CoL_2^{2+}** under N_2 atmosphere at different scan rates in DMF / 0.1 M Bu_4NPF_6. Glassy carbon disk working electrode, platinum wire counter electrode, and silver wire quasi-reference electrode.

(Bottom) Plot of reductive peak currents of the first reduction (red circles) and third reduction (black squares) from cyclic voltammograms versus the square root of the scan rate.

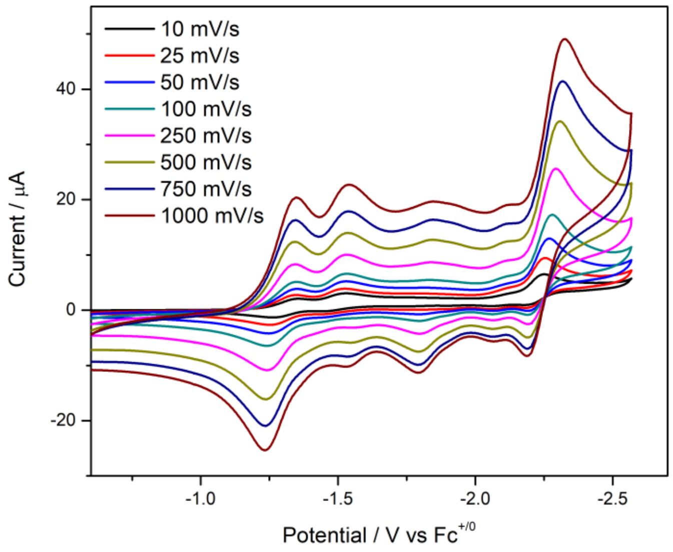

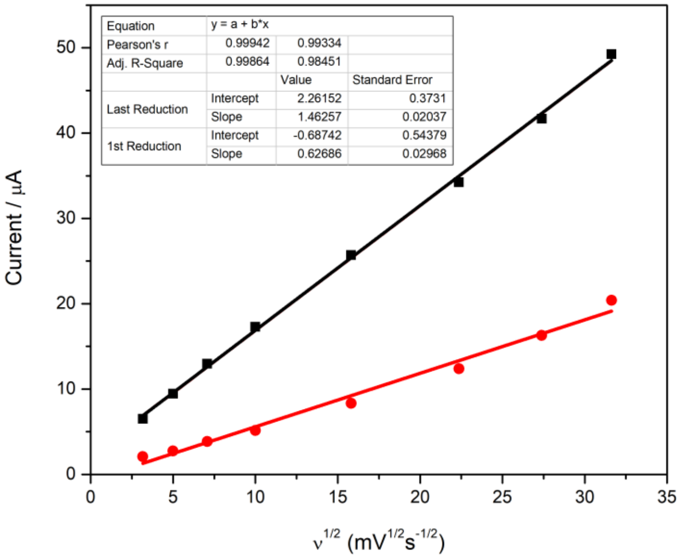

Fig. S7. (Top) CVs of 0.5 mM **NiL_2^{2+}** under N_2 atmosphere at different scan rates in DMF / 0.1 M Bu_4NPF_6. Glassy carbon disk working electrode, platinum wire counter electrode, and silver wire quasi-reference electrode.

(Bottom) Plot of reductive peak currents of the first reduction (red circles) and last reduction (black squares) from cyclic voltammograms versus the square root of the scan rate.

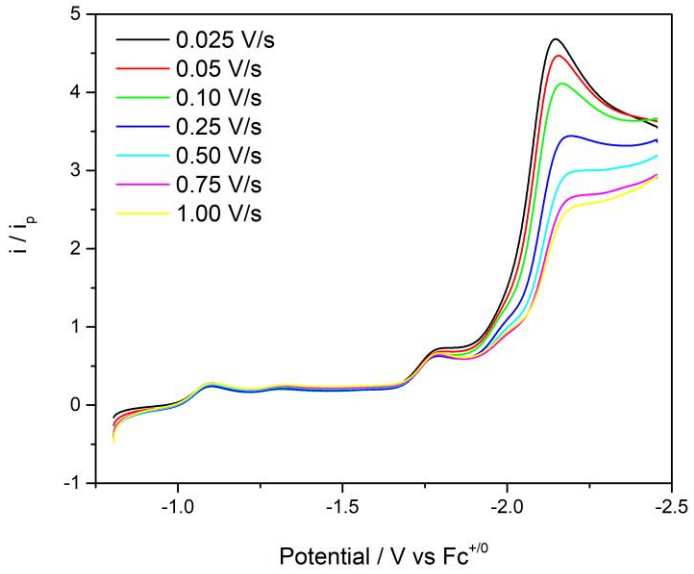

Fig. S8. Normalized linear sweep voltammograms (i_{cat}/i_p) of 0.5 mM **CoL_2^{2+}** in CO_2-saturated DMF / 0.1 M Bu_4NPF_6 containing 5% H_2O at different scan rates. Glassy carbon disk working electrode, platinum wire counter, and silver wire quasi-reference electrode.

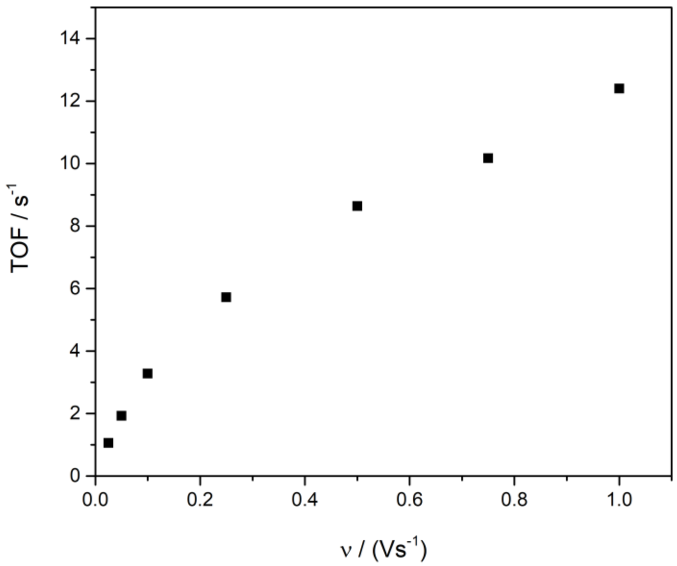

Fig. S9. Plot of turnover frequency (TOF) versus scan rate (calculated from **Fig. S8**).

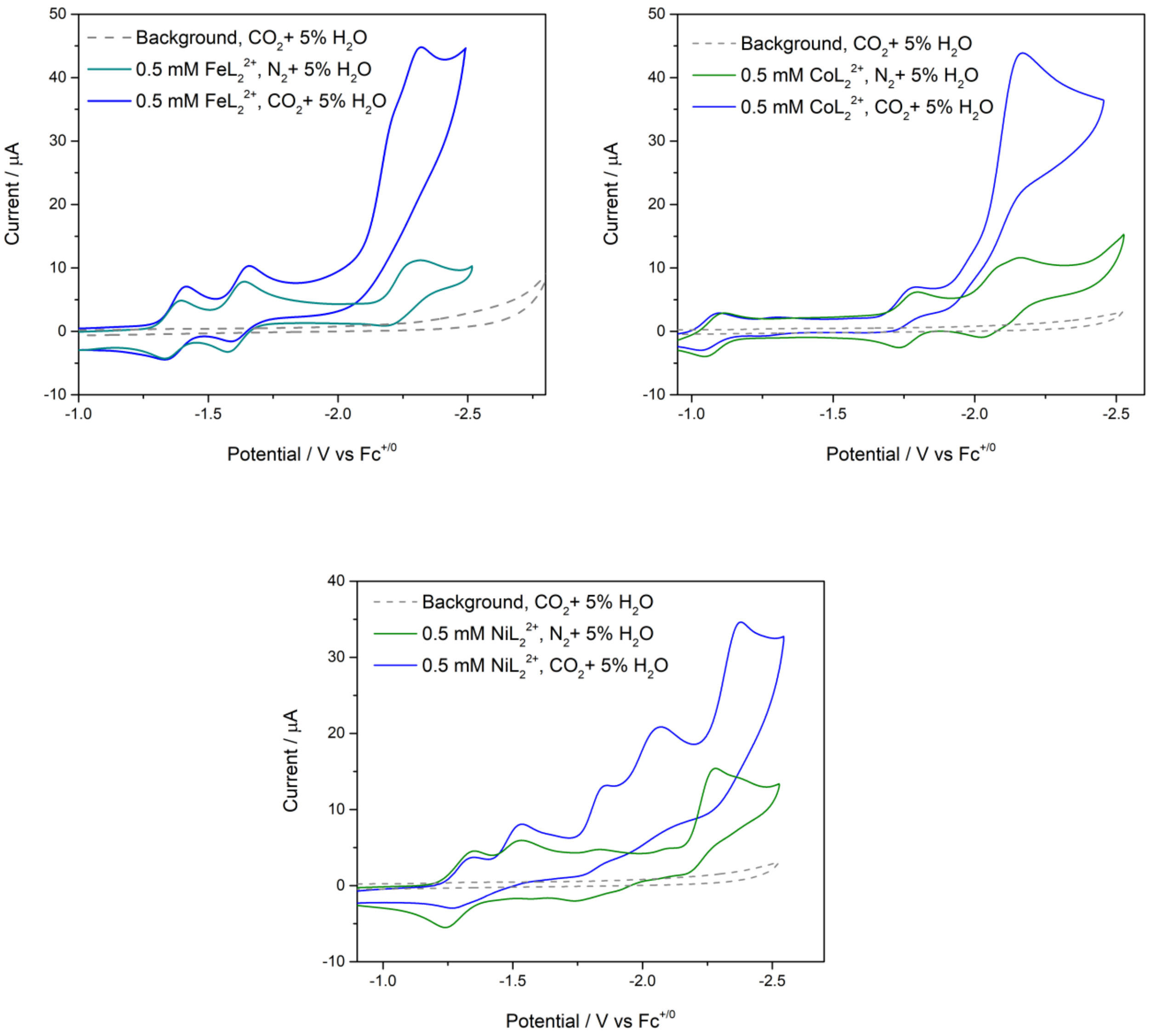

Fig. S10. CVs of 0.5 mM $\mathbf{FeL_2^{2+}}$, $\mathbf{CoL_2^{2+}}$, and $\mathbf{NiL_2^{2+}}$ under N_2 (green line) and CO_2 atmosphere (blue line) in DMF / 0.1 M Bu_4NPF_6 containing 5% H_2O. The background is shown as a dashed line under the same conditions in the absence of metal complex. Scan rate = 100 mV/s; glassy carbon disk working electrode, platinum wire counter electrode, and silver wire quasi-reference electrode.

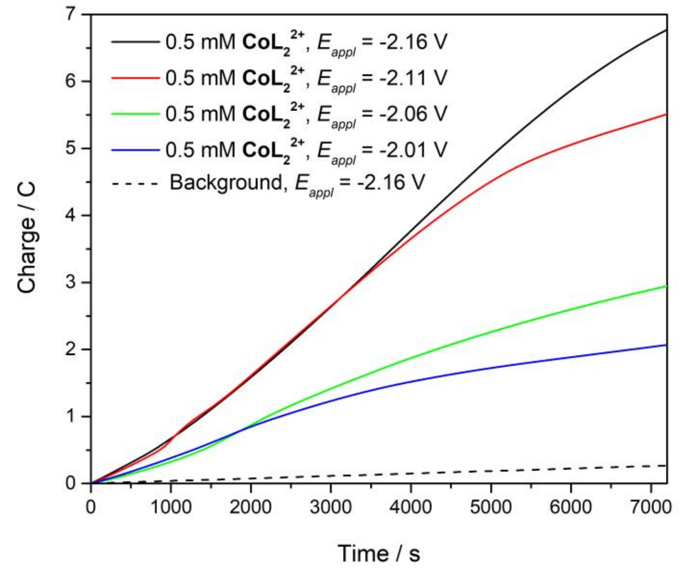

Fig. S11. Representative charge versus time plots from controlled potential electrolyses of $\mathbf{CoL_2}^{2+}$ at different applied potential. 0.5 mM $\mathbf{CoL_2}^{2+}$ in CO_2-saturated DMF/0.1 M Bu_4NPF_6 solutions containing 5% H_2O. Background CPE was done in the absence of $\mathbf{CoL_2}^{2+}$ at E_{appl} = -2.16 V. Glassy carbon rod working electrode, platinum wire counter electrode, and silver wire quasi-reference electrode.

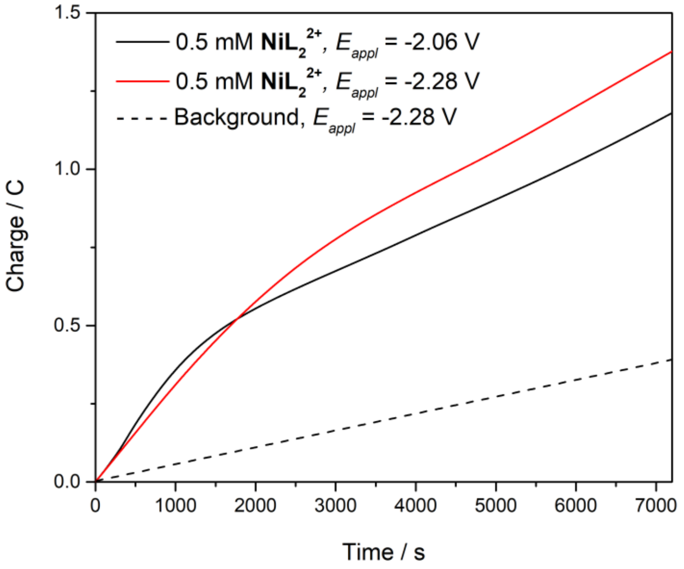

Fig. S12. Representative charge versus time plots from controlled potential electrolyses of 0.5 mM $\mathbf{NiL_2}^{2+}$ at different applied potentials in CO_2-saturated DMF / 0.1 M Bu_4NPF_6 solutions containing 5% H_2O. Background CPE was done in the absence of $\mathbf{NiL_2}^{2+}$ at E_{appl} = -2.28 V. Glassy carbon rod working electrode, platinum wire counter electrode, and silver wire quasi-reference electrode.

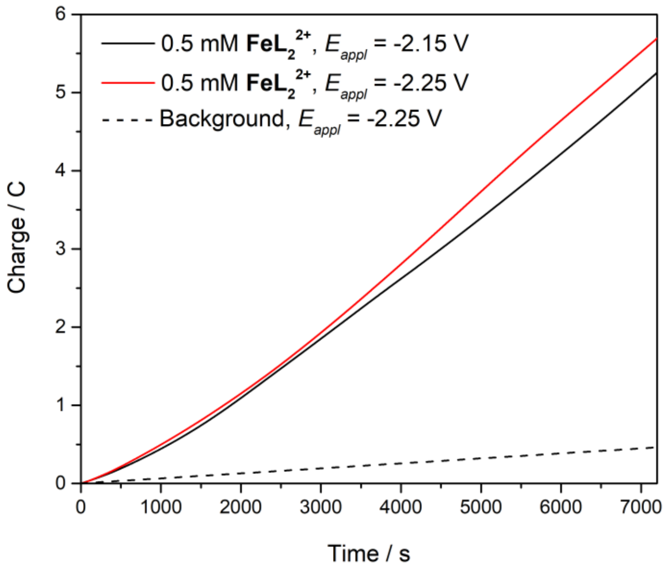

Fig. S13. Representative charge versus time plots from controlled potential electrolyses of 0.5 mM $\mathbf{FeL_2}^{2+}$ at different applied potentials in CO_2-saturated DMF / 0.1 M Bu_4NPF_6 solutions containing 5% H_2O. Background CPE was done in the absence of $\mathbf{FeL_2}^{2+}$ at E_{appl} = -2.25 V. Glassy carbon rod working electrode, platinum wire counter electrode, and silver wire quasi-reference electrode.

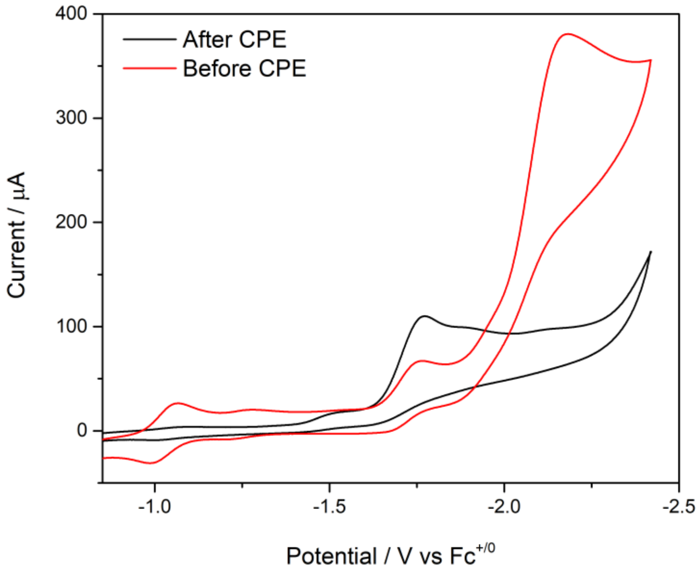

Fig. S14. Representative CVs before and after a controlled potential electrolysis (CPE) experiment of 0.5 mM $\mathbf{CoL_2}^{2+}$ in CO_2-saturated DMF / 0.1 M Bu_4NPF_6 solution containing 5% H_2O. Glassy carbon rod working electrode, platinum wire counter electrode, and silver wire quasi-reference electrode.

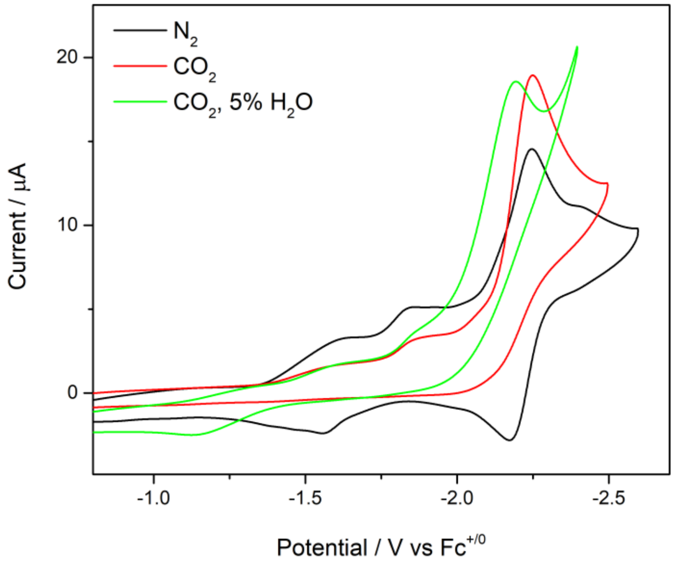

Fig. S15. Cyclic voltammograms of **ZnL_2^{2+}** at 0.5 mM concentrations in DMF / 0.1 M Bu_4NPF_6 under N_2 (black), CO_2 (red) and CO_2 with 5% H_2O (green). Scan rate = 100 mV/s; glassy carbon disk working electrode, platinum wire counter electrode, and silver wire quasi-reference electrode.

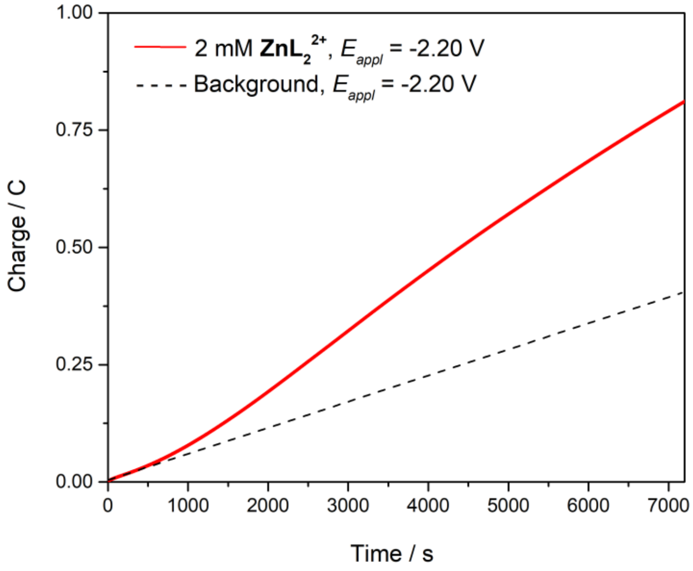

Fig. S16. Charge versus time plots from controlled potential electrolyses of 2 mM **ZnL_2^{2+}** at E_{appl} = -2.2 V in CO_2-saturated DMF / 0.1 M Bu_4NPF_6 solution containing 5% H_2O. Background CPE was done in the absence of **ZnL_2^{2+}** at E_{appl} = -2.20 V. Glassy carbon rod working electrode, platinum wire counter electrode, and silver wire quasi-reference electrode.

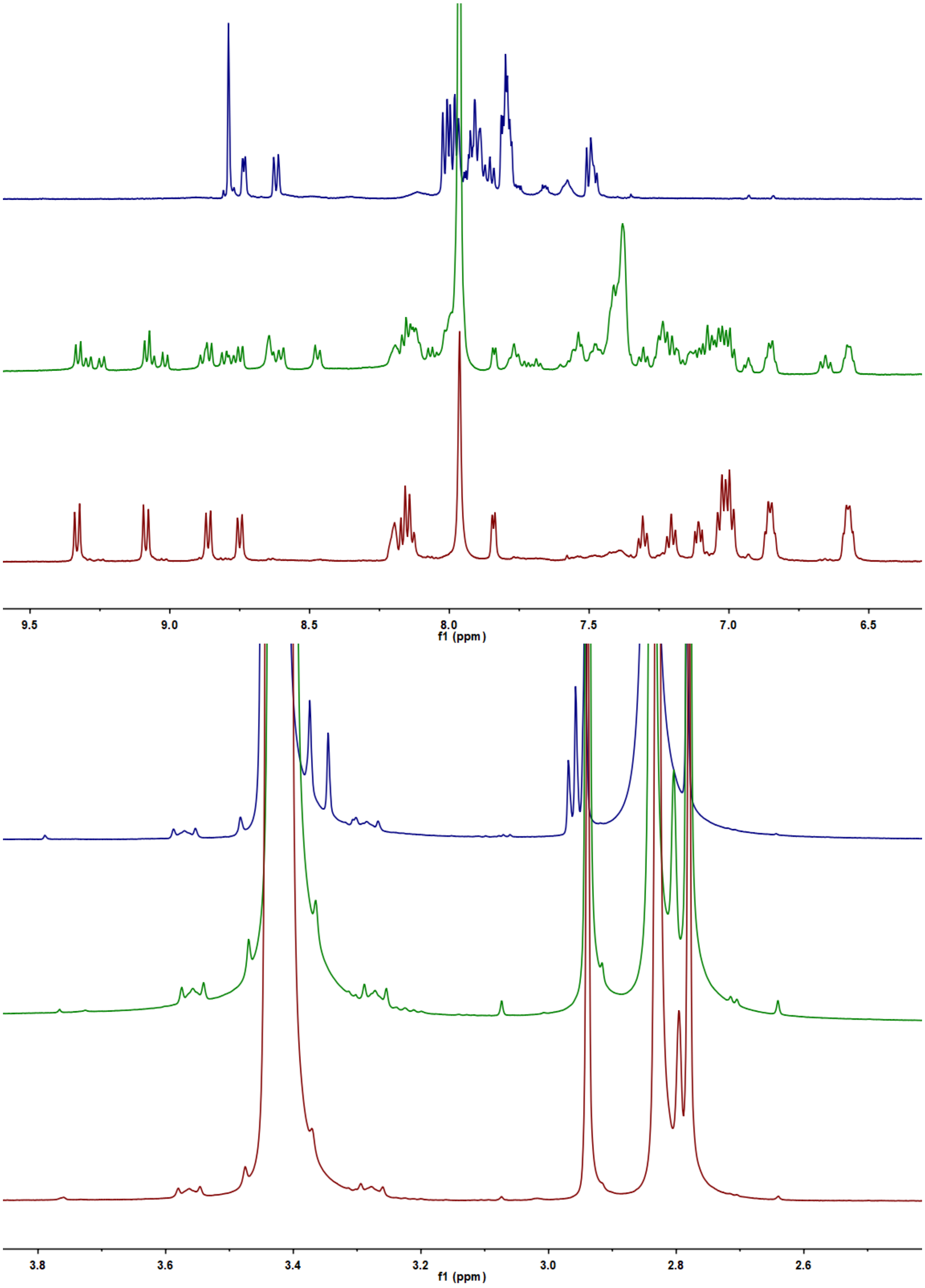

Fig. S17. ^{1}H NMR spectra (taken in acetone-d_6) of the electrolysis solution of $\mathbf{ZnL_2}^{2+}$ before electrolysis (maroon), after electrolysis (green), and after reacting with CH_3I (blue). The CPE was run for 3 hours and then excess CH_3I was added to the solution which was stirred for 1 hour. The solutions were dried under high vacuum before taking the NMRs.

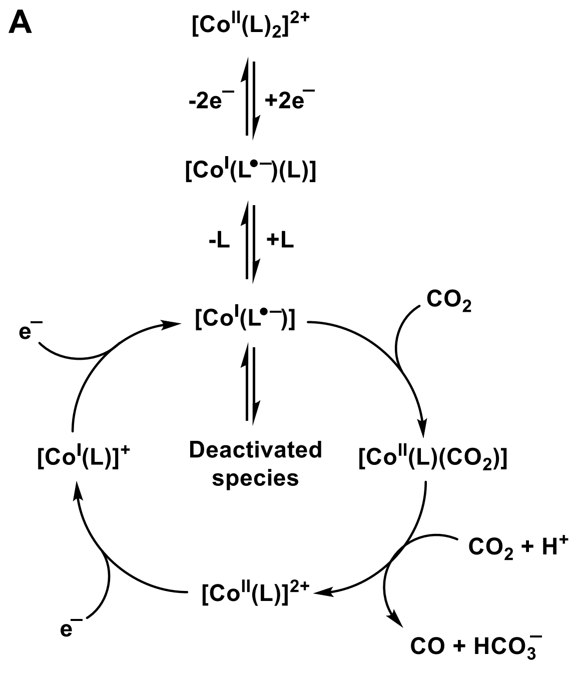

Fig. S18. Proposed mechanisms for electrocatalytic CO_2 reduction (A) and H^+ reduction (B) by the $\mathbf{CoL_2}^{2+}$ complex.
